# Diet-induced obesity induces oxidative stress and enhances H3K4me3 levels, driving nonresolving inflammation and myelopoiesis in hematopoietic stem and progenitor cells

**DOI:** 10.1093/jimmun/vkaf156

**Published:** 2025-08-01

**Authors:** Kentaro Takahashi, Julia Drolet, Jinghua Liu, Jasmine R Jackson, Muthusamy Thiruppathi, Milie Fang, Jack Rogers, Ian Davis, Giamila Fantuzzi, Elizaveta V Benevolenskaya, Timothy J Koh, Norifumi Urao

**Affiliations:** Department of Pharmacology, State University of New York Upstate Medical University, Syracuse, NY, United States; Department of Pharmacology, State University of New York Upstate Medical University, Syracuse, NY, United States; Department of Pharmacology, State University of New York Upstate Medical University, Syracuse, NY, United States; Department of Pharmacology, State University of New York Upstate Medical University, Syracuse, NY, United States; Department of Kinesiology and Nutrition, University of Illinois at Chicago, Chicago, IL, United States; Department of Kinesiology and Nutrition, University of Illinois at Chicago, Chicago, IL, United States; Department of Pharmacology, State University of New York Upstate Medical University, Syracuse, NY, United States; Department of Pharmacology, State University of New York Upstate Medical University, Syracuse, NY, United States; Department of Kinesiology and Nutrition, University of Illinois at Chicago, Chicago, IL, United States; Department of Biochemistry and Molecular Genetics, University of Illinois at Chicago, Chicago, IL, United States; Department of Kinesiology and Nutrition, University of Illinois at Chicago, Chicago, IL, United States; Department of Pharmacology, State University of New York Upstate Medical University, Syracuse, NY, United States

**Keywords:** epigenetics, hematopoietic stem, myelopoiesis, obesity, oxidative stress, progenitor cells

## Abstract

Diet-induced obesity leads to dysregulated myelopoiesis and nonresolving inflammation. Such dysregulation could involve epigenetic reprogramming, which can induce long-term changes in hematopoietic stem and progenitor cells (HSPCs). However, whether and how obesity-dysregulated HSPCs impact myelopoiesis in response to tissue injury are not fully understood. Here, we tested the hypothesis that obesity induces oxidative stress and histone H3 lysine-4 trimethylation (H3K4me3) in HSPCs, programming enhanced myelopoiesis and persistent inflammation, leading to impaired tissue recovery. Transfer of bone marrow HSPCs from high-fat diet–induced obese mice (HFD-HSPCs) to lean recipients was sufficient to drive nonresolving myelopoiesis and impaired tissue recovery from hindlimb ischemia. HFD-HSPCs exhibited increased oxidative stress that drives elevated H3K4me3 and reduced KDM5 demethylase activity. CUT&Tag (cleavage under targets and tagmentation) analysis revealed H3K4me3 enrichment at cell cycling regulating E2F targets during myeloid differentiation and *Tlr4* gene promoter in HFD-HSPCs. Such enrichment is associated with increased TLR4-driven myelopoiesis in vitro, increased inflammatory myelopoiesis during hindlimb ischemia, and myeloid bias after serial transplantations in lean recipients. Knockout of KDM5A, an H3K4me3 demethylase and negative regulator of E2F activity, increased H3K4me3 in HSPCs, enhanced TLR4-driven myelopoiesis in vitro, and increased myelopoiesis in vivo. Furthermore, cyclosporine A treatment in HSPCs ex vivo reduced oxidative stress, normalized H3K4me3 levels, and mitigated enhanced myelopoiesis in HSPCs in HFD mice. Our findings suggest that oxidative stress by diet-induced obesity enhances H3K4me3 levels and increases myelopoiesis in HSPCs, leading to persistent inflammation and impaired recovery from hindlimb ischemia.

## Teaser

The Editors have selected this article as a highlight of the issue.

## Introduction

Obesity is a major global health challenge, predisposing individuals to chronic inflammatory diseases, metabolic dysfunction, and increased cardiovascular risk.^1^ Mounting evidence, using mouse models of obesity such as high-fat diet (HFD)–induced obesity, suggests that obesity-driven alterations in the hematopoietic system contribute to sustained inflammation and tissue injury.^2–7^ Hematopoietic stem and progenitor cells (HSPCs) continuously replenish immune cells, and studies indicate that inflammatory cues can reprogram HSPCs to promote myeloid-biased differentiation^8–10^ and that HFD-induced obesity indeed leads to long-term alterations in HSPC fitness through oxidative sress.^11^

Epigenetic modifications, particularly histone methylation, have emerged as key regulators of trained immunity, influencing long-term hematopoietic memory following inflammatory/metabolic stimuli^10^^,^^12–14^ and monocytes/macrophages during wound healing.^6^^,^^7^^,^^15^ Specifically, histone H3 lysine 4 trimethylation (H3K4me3) is linked to proinflammatory gene activation in myeloid cells and wound macrophages in HFD-induced immune programming.^6^^,^^15^^,^^16^ H3K4me3 is also highly associated with the activating state of chromatin specifying cell identity.^17^ However, whether H3K4me3 drives enhanced myelopoiesis in diet-induced obesity has yet to be determined.

In this study, we demonstrate that HSPCs from HFD-induced obese mice (HFD-HSPCs) is sufficient to drive persistent inflammation and impaired tissue recovery in lean recipients following ischemic injury, providing direct evidence that obesity reprograms the hematopoietic system toward a proinflammatory and nonresolving phenotype. HFD-HSPCs exhibited increased oxidative stress and elevated H3K4me3 levels in with reduced activity of the H3K4me3 demethylase KDM5A, suggesting a mechanistic link between metabolic stress and persistent epigenetic alterations. Indeed, KDM5A gene knockout (KO) and HFD-HSPCs showed promoted myelopoiesis. Furthermore, we show that cyclosporine A (CsA) treatment reduces oxidative stress, normalizes H3K4me3 levels, and restores HSPC function, providing a potential therapeutic strategy to counteract obesity-induced immune dysfunction. Finally, epigenomic profiling via CUT&Tag reveals differential H3K4me3 enrichment at key inflammatory and myeloid differentiation loci, including *Tlr4* and E2F targets, which may contribute to the heightened inflammatory responsiveness of HSPCs for nonresolving myelopoiesis in HFD-induced obesity.

## Materials and methods

### Animals

Animal protocols used for experiments were approved by the Institutional Animal Care and Use Committees of Upstate Medical University and the University of Illinois at Chicago, and animals were cared for according to National Institutes of Health guidelines for the care and use of laboratory animals under protocol number #468. All animals were group-housed in a temperature-controlled facility with a 12-h light/dark cycle. C57BL/6J male mice (strain #000664) at the age of 3 to 4 wk were purchased from the Jackson Laboratory and fed a high-fat diet (HFD) (60% kcal fat; Research Diets; D12492) or low-fat control diet (LFD) (10% kcal fat; Research Diets; D12450B) with the same protein content as the HFD for 16 to 20 wk. B6 45.1 mice (B6.SJL-*Ptprc^a^ Pepc^b^*/BoyJ, strain #002014) and EGFP mice (C57BL/6-Tg(CAG-EGFP)131Osb/LeySopJ, strain #006567) were purchased from the Jackson Laboratory, and house-bred male mice at similar age (within 1 mo) fed HFD or LFD for 20 wk were used as donors of bone marrow (BM) transplantation or adoptive transfer experiments. All the comparisons between HFD- and LFD-fed mice were made strictly within the same cohort of animals. Eight-week-old male C57BL/6J were used as recipients of BM transplantation. Tamoxifen-inducible *Kdm5a* KO mice, crossbred B6.129S6(Cg)-Kdm5a^tm1Kael^/J (RRID: IMSR_JAX: 008571) with B6.129-Gt(ROSA)26Sor^tm1(cre/ERT2)Tyj^/J (RRID: IMSR_JAX: 008463), were house bred from breeders given by the laboratory of Dr. William Kaelin Jr. The gene KO was induced by intraperitoneal tamoxifen (75 mg/kg body weight) once every 24 h for a total of 5 consecutive days. Genotyping was performed by Transnetyx. All the comparisons between Cre+ and Cre− mice were made strictly within the littermates.

### Mouse tissue injury models

Femoral artery ligation at the levels of the inguinal ligament and the bifurcation of the deep femoral artery, which was followed by resection of the artery in between, was performed as we previously reported and characterized.^18^^,^^19^ The tibialis anterior muscle was enzymically digested and the total cell suspension was analyzed by flow cytometry.^20^ Magnetic resonance imaging was performed in the Research Resource Core MRI core at the University of Illinois at Chicago. A live mouse was scanned by 9.4T magnetic resonance imaging under isoflurane anesthesia. The acquired T2-enhanced images with a voxel size of 25 μm were processed, and 3-dimensional rendering/segmentation was performed by a DICOM viewer software, OsiriX 64-bit or MD (Pixmeo).

Dorsal skin biopsies were performed like we showed.^21^^,^^22^ Two 8-mm-diameter full-thickness excisional skin wounds were made on the back of each mouse with a dermal biopsy punch under anesthesia and analgesia. The wounds were covered with Tegaderm (3M) to keep the wounds moist until day 7 after wounding. At indicated time points, mice were euthanized by isoflurane asphyxiation and bilateral thoracotomy, and the tissues were harvested for analysis.

### Flow cytometry

Cell surface and intracellular staining was performed as described.^20^ Primary anti-mouse Abs included lineage biotin (NK1.1 (PK136), CD11b (M1/70), CD19 (MB19-1), B220 (RA3-6B2), CD3 (145-2C11), TER-119 (TER-119), CD8a (53-6.7), CD4 (GK1.5), and Gr-1 (RB6-8C5)), Sca-1 Pacific Blue (D7) or PECy7 (D7) or PerCP (D7), c-Kit APC-eFluor 780 (2B8), or AF647 (2B8), CD19 Cy5PE (MB19-1), B220 APC (RA3-6B2,), CD115 PE or BV605 (AFS98), CD135 PE (A2F10, dilution 1/50), CD150 PE (mShad150), CD48 BV711 (HM48–1), FcγR BV510 (93), CD14 FITC (Sa2–8), CD45.1 AF647 (A20), CD45.2 APC or BV421 (104), MHCII APC, Ly6C BV785 or FITC, Ly6G BV421, or PE-Dazzle594. Secondary reagents were streptavidin-PECy5, streptavidin-PECy7, or streptavidin-BV421. Intracellular staining for H3K4me3, H3K4me1, H3K27ac, and HIF-1α, EdU incorporation and annexin V staining was performed as we have previously described.^18^ For intracellular staining, cells were fixed with 4% paraformaldehyde in phosphate-buffered saline for 15 min at room temperature, followed by permeabilization with 0.1% Triton X-100 for 15 min. After washing, cells were incubated with a rabbit anti-mouse H3K4me3, H3K4me1, H3K27ac or HIF-1α, or control IgG primary antibody (1:200; Abcam) in FACS buffer for 30 min at 4 °C. Following another wash, cells were stained with an anti-rabbit IgG secondary antibody conjugated to AF647 (1:200) for 30 min at 4 °C. Finally, cells were washed, resuspended in FACS buffer, and analyzed by flow cytometry.

For EdU, mice were injected intraperitoneally with 600 μg EdU (APExBIO; B8337) or phosphate-buffered saline, and 4 h after the injection BM was isolated. Cells were stained for surface markers followed by intracellular staining with anti-EdU antibodies using the EdU flow cytometry kit according to the manufacturer’s instructions (APExBIO; K1078).

TLR4 expression levels and oxidative stress measurement were analyzed in unfractionated BM samples stained with Lin^−^Sca1^+^cKit^+^ (LSK) markers, in combination with metabolic inhibitors. Unfractionated BM was cultured in RPMI media with 10% fetal bovine serum or in StemPro34 media with nutrient supplement. After 4 h of incubation at 37 °C and with 5% CO_2_, the LSK marker stained cells were stained by biotinylated TLR4 antibody (BioLegend) followed by a fluorescent secondary antibody or incubated with CellROX DeepRed (5 uM for 15 min), MitoPY1 (Sigma-Aldrich; cat. SML0734, lot 063M4613V, for 15 min) or MitoTracker CMTMH2Ros (Thermo Fisher Scientific; 400 nM for 15 min). Metabolic manipulation was performed at the start of culture, and 2-DG (10 mM, Sigma-Aldrich), dimethyl succinate (10 mM, Sigma-Aldrich; W239607-SAMPLE-K), mitoTEMPO (20 μM, Sigma-Aldrich; SML0737), CsA (10 μM), or dimethyl sulfoxide control supplemented the culture media.

Tissue digestion was performed as we described^22^ using an enzyme cocktail to obtain single-cell suspensions for flow cytometry analysis. The enzyme solution was prepared by dissolving collagenase I, collagenase XI, and hyaluronidase at 5 mg/mL each, along with DNase (0.45 mg/mL) in Dulbecco’s Modified Eagle Medium containing calcium and magnesium. The enzyme solution was prewarmed to 37 °C in a heat block prior to use. Tissue samples were first weighed to determine cell yield per tissue weight. The samples were enzymatically digested for a total of 60 min with sequential mechanical disruption to enhance dissociation. At the start of digestion (0 min), the tissue was finely minced with scissors. At 15 min, the partially digested tissue was triturated by passing through an 18G needle 10 times using a 1 mL syringe. This process was repeated at 30 min with a 23G needle and at 45 min with a 26G needle. At the end of the digestion (60 min), the suspension was pipetted vigorously to ensure complete dissociation. The resulting cell suspension was filtered through a 70 μm mesh into a 50 mL tube, and the mesh was rinsed with 5 mL of FACS buffer. Cells were pelleted by centrifugation at 400 *g* for 5 min, resuspended in 1 mL of FACS buffer, and further filtered through a 40 μm mesh. The final cell count was determined using a hemocytometer without dilution, employing acridine orange/propidium iodide staining to assess viability.

Flow cytometry was performed on a 4 laser 18 detector LSRFortessa (BD Biosciences) or a 5 laser Cytek Aurora. Data were analyzed with FlowJo software v10.8 (BD Biosciences).

### HSPC isolation

Bones from LFD or HFD mice, or EGFP (C57BL/6-Tg(CAG-EGFP)131Osb/LeySopJ) were dissected, including the femur, tibia, iliac, and brachial bones from the legs and arms. BM cells were collected either with centrifugation at 10,000 *g* for 15 s or flushing with FACS buffer, and purified by Ficoll separation with Histopaque-1119 (Millipore, Sigma-Aldrich). Note that CsA treatment (50 µg/mL) was performed immediately after flushing the BM cells at 4 °C. Cells were stained with unconjugated rat anti-mouse lineage-specific antibodies (Ter-119, Mac1, Gr-1, B220, CD5, CD3, CD4, CD8, and CD127; BioLegend), followed by incubating with Dynabeads anti-rat IgG (Thermo Fisher Scientific). Unbound cells including HSPCs were collected after magnetic sorting. Cells were then stained with goat anti-rat PE-Cy5 secondary antibody (Thermo Fisher Scientific) for lineage-specific primary antibodies, and c-kit–APCeFluor780 (Thermo Fisher Scientific), Sca1-PB (BioLegend), and CD150-PE (BioLegend) antibodies. Cell sorting was performed on a FACSAria III cell sorter (BD). Data analysis was performed using FlowJo software. In the CsA-treated group, HSPCs were isolated in buffers containing 50 µg/mL. The sorted cells were collected in RPMI supplemented with 30% fetal bovine serum for further analysis and downstream applications.

### HSPC culture

We performed HSPC culture immediately after their sorting as described.^22^ Sorted cells were cultured in StemPro34 media (Thermo Fisher Scientific) supplemented with its nutrient supplement, 2 mM L-glutamine, 100 U/mL penicillin and 100 mg/mL streptomycin, and mouse SCF (PeproTech) (20 ng/mL). For stimulation, lipopolysaccharide (LPS) (InvivoGen; 10 µg/mL) or LPS (InvivoGen; 1 µg/mL) +Pam3CSK4 (InvivoGen; 100 ng/mL) and M-CSF (PeproTech; 1 µg/mL) was added to the culture media when starting cell culture at 37 °C with 5% CO_2_ in a round-bottom 96-well plate (3,000–5,000 cells per well). Like a previous study,^23^ CsA treatment (50 μg/mL) was performed immediately after flushing the BM cells at 4 °C as described in the cell isolation protocol and was continued until starting the cell culture. We confirmed there was no change in the LSK population rate by CsA treatment. After a 3- or 4-d culture, total cells in the cell colony were detached, stained with antibodies (Ly6C FITC, F4/80 PE, CD11b APC), and analyzed by flow cytometry. The yield of myeloid cells was calculated by % indicated myeloid population, total cell event numbers and the average cell number in control (nonstimulated). The average of triplicate cultures was used to represent 1 sample source. For JARID (KDM5) or H3K4me3 methyltransferase activity measurement, Epigenase JARID Demethylase Activity/Inhibition Assay Kit (EpigenTek; P-3082) or EpiQuik Histone Methyltransferase Activity/Inhibition Assay Kit (H3K4) (EpigenTek; P-3002) was used to measure enzymic activity in isolated nucleus following EpiQuik Nuclear Extraction Kit (OP-0002-1). The nucleus was isolated Lin^−^-enriched HSPCs cultured in StemPro34 media for 2 h.

### CUT&Tag assay

Samples for cleavage under targets and tagmentation (CUT&Tag) analysis were prepared as follows with courtesy communication with Dr. Steven Henikoff (Fred Hutchinson Cancer Center). Ice-cold NE1 buffer (100 µL) was added directly to the sorted cells (10^4^ LSK or granulocyte-macrophage progenitor [GMP] cells). Subsequently, the cells were incubated on ice for 10 min to ensure proper nuclear isolation. Following incubation, cells were centrifuged at 600 *g* for 3 min using a swinging bucket rotor at room temperature, and the supernatant was carefully removed. The pelleted cells were then resuspended in 100 µL of ice-cold NE1 buffer with gentle vortexing to ensure proper suspension. Nuclei were checked using a cell counter slide, with staining performed using acridine orange/propidium iodide, and the dead cell count (red cells) was noted. Optionally, nuclei could be slowly frozen by aliquoting with 10% dimethyl sulfoxide, mixed well, and placed in a Mr. Frosty container (Thermo Fisher Scientific) filled to the line with isopropanol for storage at −80°C overnight.

Concanavalin A (ConA)–coated beads were prepared and employed to bind nuclei in the following manner. ConA bead slurry was transferred at a rate of 10 µL per sample into 100 µL per sample of Binding buffer in 1.5 mL tubes and thoroughly mixed by pipetting. The tubes were placed on a magnet stand to clear for 30 s to 2 min. After the complete removal of the liquid, the tubes were removed from the magnet stand, and an additional 100 µL per sample of Binding buffer was added and mixed. The tubes were once again placed on the magnet stand to clear, and the liquid was withdrawn, followed by the resuspension of the beads in 10 µL per sample of Binding buffer. Cells were then mixed with ConA beads by combining 300 µL of cell suspension (resuspended in Wash150 buffer) with 10 µL of ConA beads in low-retention 1.5 mL tubes at room temperature for 10 min. Tubes were placed on a magnet stand to clear, and the liquid was withdrawn.

Primary and secondary antibodies were used to bind to the nuclei-bead complex. Cells were resuspended in 50 µL of Antibody buffer (consisting of 500 µL wash buffer, 10 µL 5% digitonin, and 1.667 µL 30% bovine serum albumin), followed by the addition of 0.5 µL of primary antibody (H3K4me3 or IgG) (1:100). The mixture was gently vortexed and incubated overnight at 4 °C on a rotator.

On the following day, tubes were placed on the magnet stand to clear, and the liquid was withdrawn. A secondary antibody, diluted 1:100 in Digitonin150 buffer, was added at 100 µL per sample while gently vortexing to dislodge beads. Tubes were rotated at room temperature for 30 min. After a quick spin, tubes were placed on a magnet stand to clear, and the liquid was withdrawn. While still on the magnet stand, 500 µL of Digitonin buffer was carefully added and withdrawn. This process was repeated twice for a total of 2 washes.

To bind the pAG-Tn5 adapter complex, CUTANA pAG-Tn5 was mixed in the 300-wash buffer to a final concentration of 1:20. The mixture was then added to samples, with 50 µL per sample being squirted in while vortexing to dislodge beads. After a quick spin, tubes were placed on a rotator at room temperature for 1 h. Tubes were placed on a magnet stand to clear, and the liquid was withdrawn. While still on the magnet stand, 500 µL of Digitonin300 buffer was added and withdrawn. This process was repeated, and the liquid was withdrawn.

After the previous steps, the bead/nuclei pellet was resuspended in 300 µL of tagmentation solution while vortexing or inverting by rotation. Following a quick spin, incubation was carried out at 37 °C for 1 h in a polymerase chain reaction (PCR) cycler with a heated lid. The tagmentation process was stopped by the addition of EDTA, SDS, and proteinase K. Subsequent steps involved DNA extraction and purification. DNA samples were subjected to PCR using NEBNext HiFi 2× PCR Master Mix (New England Biolabs) and specific primers. Post–PCR clean-up involved SPRI bead purification followed by ethanol washes and elution in Tris-HCl buffer. Fragmentation of DNA was checked by BioAnalyzer (Agilent).

### Analysis of CUT&Tag data

Paired-end FASTQ files were processed using the nf-core-cutandrun pipeline (v3.1).^24^^,^^25^ Adapter sequences were removed with Trim Galore. Reads were aligned with Bowtie2^26^ to GRCm39 (National Center for Biotechnology Information, release 109) and duplicates were removed for only control samples using Picard. Peaks were called with SEACR (normalization mode: CPM).^27^ Peaks were defined as regions where at least 1 of the target samples significantly exceeds its matched IgG control for a given genomic region. Consensus peaks were merged across all samples using BEDTools^28^ and annotated to the nearest gene transcriptional start site using HOMER.^29^ Peak counts were generated using featureCounts.^30^ Peaks were visualized on an Integrative Genome Viewer^31^ plot using the igv.js library.^32^

Differential peak analysis was performed comparing the groups. The comparison was performed with the DESeq2 R package,^33^ which tests for differential abundance based on a model using the negative binomial distribution. Differential peak analysis results account for experimental covariates such as batch and paired samples.

Functional annotation analysis of consensus peaks was performed using the annotatePeak function from the ChIPseeker R package.^34^^,^^35^ Peaks were annotated with respect to their genomic location relative to transcription start sites, with a region of ∼3,000 base pairs upstream and 3,000 base pairs downstream of the transcription start sites. Transcript-related features, in TxDb format, from the UCSC Genome Browser database^36^ for NCBI_GRCm39_release109_chr were used for peak annotation.

Gene set enrichment analysis (GSEA) using the fgsea R package and the fgseaMultilevel() function (doi:10.1101/060012). The log_2_ fold change from the H3K4me3 GMP vs H3K4me3 LSK differential expression comparison was used to rank genes. Hallmarks gene set collection from the Molecular Signatures Database (MSigDB)^37^^,^^38^ was curated using the msigdbr R package (https://CRAN.R-project.org/package=msigdbr). Prior to running GSEA, the list of gene sets was filtered to include only gene sets with between 5 and 1,000 genes. The bar plot shows the GSEA results for each tested gene set with a *P* value ≤ 0.01 (Fig. 4C). The y-axis shows the normalized enrichment score (NES) from GSEA, which represents the magnitude of enrichment as well as the direction. A positive NES indicates more enrichment in the first group, while a negative NES indicates more enrichment in the second group. Gene sets are ordered along the x-axis by NES value. Analysis was performed using the Pluto (https://pluto.bio) CUT&RUN pipeline.

To curate a gene list based on chromatin accessibility (assay for transposase-accessible chromatin [ATAC] signals) involved in determining an hematopoietic stem cell (HSC), myeloid, or lymphoid lineage under normal conditions, we conducted a literature review.^10^^,^^39–42^ In total, 340 lineage-specific genes (101 HSC lineage genes, 165 myeloid lineage genes, and 66 lymphoid lineage genes) were identified using this method (Table S5). Much of the data used in this analysis were derived from the figures of the papers used in the literature review. However, each study used a different set of control conditions, which were individually identified by reviewing the text for each paper.

### Quantitative reverse-transcriptase PCR

Total RNA was extracted from frozen 10^3^ cells aliquots using TRIzol (Invitrogen) with linear acrylamide (Ambion) according to the manufacturer’s manual. Residual genomic DNA was removed by treatment with DNase I (Sigma-Aldrich). First-strand complementary DNA synthesis was carried out using SuperScript VILO complementary DNA Synthesis Kit (Invitrogen) quantitative PCR was performed using Brilliant III Ultra-Fast SYBR Green qPCR Master Mix (Agilent Technologies; 600882) in duplicates. Ct values were obtained on an Agilent 8890 Real-Time PCR System, and data were generated with the comparative threshold cycle (Delta CT) method by normalizing to hypoxanthine phosphoribosyltransferase (*Hprt*). The list of primers is provided in Table S6.

### Adoptive transfer and BM transplantation

For adoptive transfer of 10^4^ LSK cells, sorted LSK cells from EGFP mice, or CD45 congenic strains were injected via retroorbital cavity into indicated lean recipients. If hindlimb ischemia was subjected, transfer was performed just after the completion of hindlimb ischemia surgery.

For BM transplantation assays, 8- to 12-wk-old C57BL/6 recipient mice were lethally irradiated (9 Gy in 1 dose) and retro-orbitally injected with 5,000 purified SLAM HSPCs (EGFP+) together with 100,000 whole BM cells obtained from C57BL/6 mice (EGFP−) as competitors. Peripheral blood was collected by retroorbital bleeding at 8 and 16 wk after transplantation and stained with specific antibodies for flow cytometry analysis. For serial transplantation, HSPCs were isolated 16 wk after the first transplantation. Lethally irradiated 8 to 12-wk-old C57BL/6 recipient mice were injected with 3,000 HSPCs (EGFP+) together with 100,000 whole BM cells obtained from C57BL/6 mice (EGFP−) as competitors.

## Results

### BM transfer from HFD-induced obesity impairs tissue recovery from ischemic injury in lean recipients

**Figure 1.**
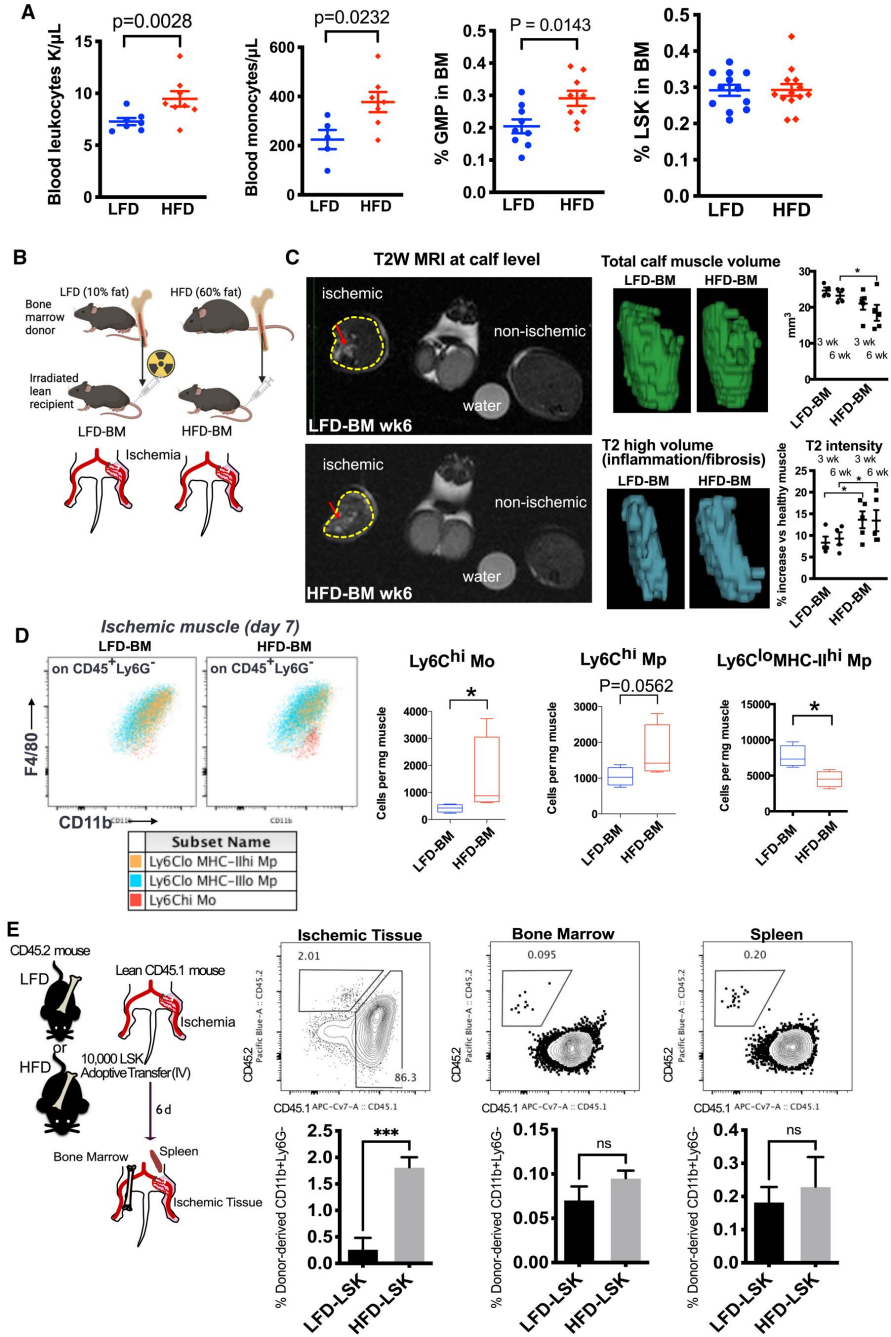
BM from HFD mice is sufficient to induce nonresolution in ischemic hindlimb muscles. (A) Blood and BM cells from mice fed either 10%cal fat LFD or 60%cal HFD were analyzed by flow cytometry to count monocytes (CD115^+^Ly6G^−^CD11b^+^), Lin^−^Sca1^+^cKit^+^ (GMP) and LSK. (B) Scheme of BM chimera mice subjected to hindlimb ischemia after reconstitution of the hematopoietic system in irradiated lean recipients. (C) Volumetric analysis of T2-weighted magnetic resonance imaging shows calf muscle volume and relative T2-high (indicative of inflammation/fibrosis, red arrow) at indicated time point after hindlimb ischemia. Water in a plastic tube was used as a control (n = 4 or 5 mice). (D) Flow cytometry analysis of enzymically digested tibialis anterior muscle on day 7 shows F4/80^lo^-Ly6C^hi^ monocytes (Mo), F4/80^hi^-Ly6C^hi^ macrophages (Mp), and F4/80^hi^-Ly6C^lo^-MHC-II^hi^ Mp (n = 4 or 5 mice). (E) Flow cytometry–sorted LSK hematopoietic stem progenitor cells from LFD and HFD mice were intravenously injected to lean CD45.1 mice immediately after hindlimb ischemia surgery. After 6 d, the ischemic tibialis anterior, BM, and spleen were analyzed by flow cytometry. The proportion of donor-derived CD45.2^+^/CD11b^+^/Ly6G^−^ monocytes/macrophages was quantified (n = 6 mice). For all panels, data are shown as the mean ± SEM. Statistical significance was determined using 1-way analysis of variance with Turkey’s multiple comparison test in panel C; and using Student’s *t* test in panels A, D, and E. *P < 0.05; *** *P* < 0.001. MRI, magnetic resonance imaging; ns, not significant; T2W, T2 weighted.

Mice fed an HFD (60 kcal%; HFD mice) exhibited significantly increased body weight (52.4 ± 4.8 g vs. 35.1 ± 4.8 g at 20 wk of age; n = 22; *P*  < 0.0001), reduced glucose tolerance,^43^ and mildly elevated fasting glucose levels (164.1 ± 27.3 mg/dL vs. 190.8 ± 35.5 SD mg/dL; n = 14; *P* = 0.035) compared with mice fed control LFD mice. Consistent with previous studies in diet-induced obesity in mice,^3^^,^^4^^,^^44^^,^^45^ our cohorts of HFD mice exhibited leukocytosis and monocytosis (CD115^+^Ly6G^−^Ly6C^+^) in the blood and increased abundance of myeloid compartments such as GMP cells (Lin^−^Sca1^−^cKit^+^FcγR^+^CD34^+^) in the BM but showed unaltered LSK HSPCs (Fig. 1A).

To test whether HSPCs in HFD-induced obese mice impair resolution of inflammation, we transplanted whole BM from HFD mice or LFD mice into LFD mice and then performed hindlimb ischemia surgery (Fig. 1B). Using T2-weighted magnetic resonance imaging, in which inflammatory tissue can be detected as areas with higher intensity, we found prolonged inflammation and larger loss of muscle volume in HFD-BM chimeras compared with LFD-BM chimeras (Fig. 1C). In flow cytometry analysis of ischemic muscle, more inflammatory Ly6C^hi^ monocytes and less anti-inflammatory Ly6C^lo^/MHC-II^hi^ macrophages were observed in HFD-BM chimeras (Fig. 1D). To study whether obesity-dysregulated HSPCs directly contributed to proinflammatory monocyte accumulation in ischemic muscle, we used adoptive transfer of sorted LSK cells in a CD45 congenic system and established in vivo fate tracking of donor HSPCs in muscle, BM, and spleen. Compared with LFD-HSPCs, we found more donor-derived monocytes/macrophages in ischemic muscle on day 6, while we did not observe differences in BM and spleen (Fig. 1E). These results suggest that HFD-induced obesity leads to long-term alteration of HSPCs that is associated with persistent inflammation and impaired tissue recovery following ischemia.

### HFD-induced obesity increases oxidative stress and H3K4me3 in HSPCs

**Figure 2.**
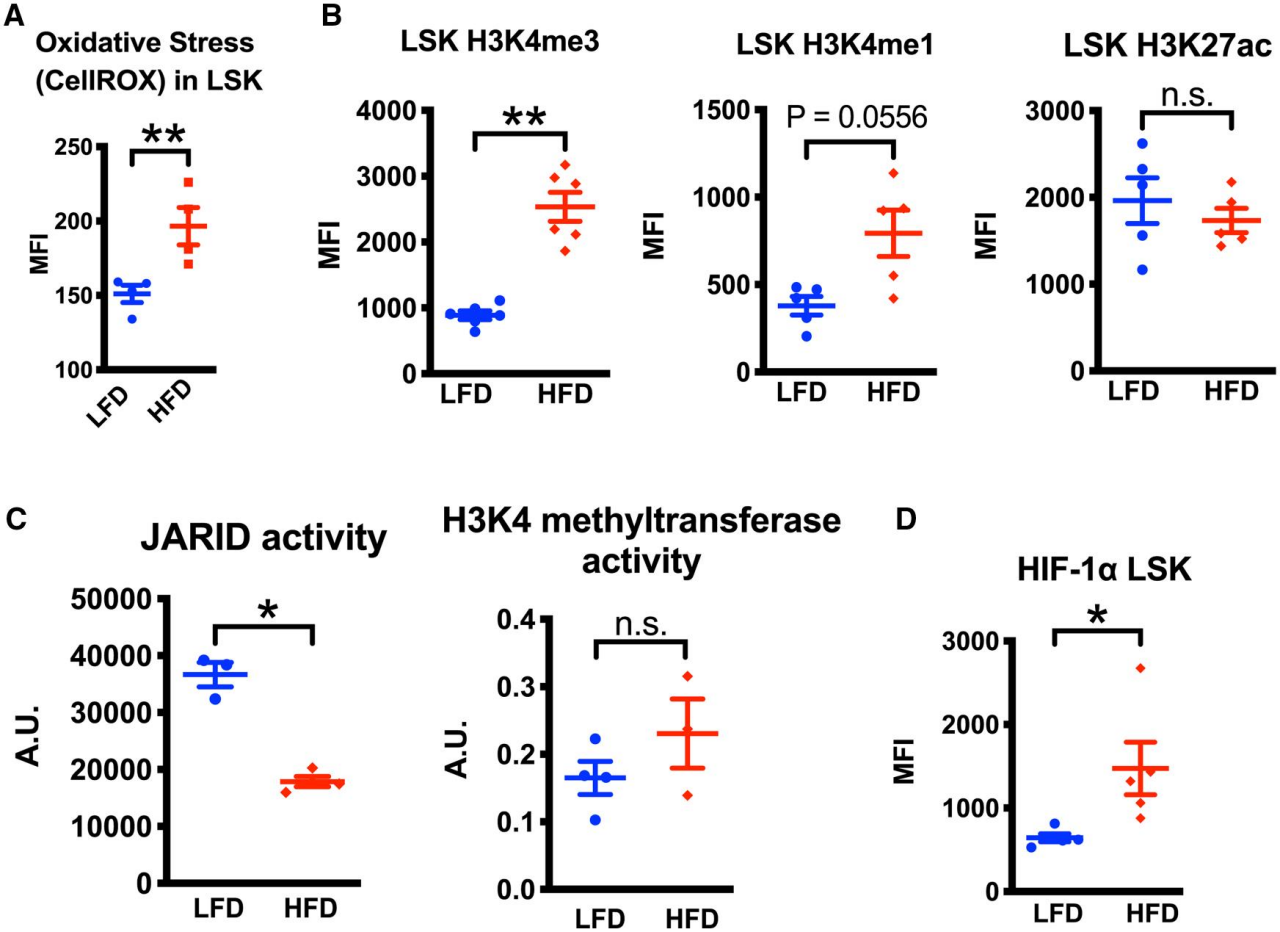
HSPCs from HFD mice exhibit increased oxidative stress and H3K4me3 levels along with decreased H3K4me3 demethylase activity. (A) Flow cytometry on LSK BM cells from LFD or HFD mice shows median fluorescence intensity (MFI) of CellRox deep red signals compared with unstained control (n = 4 mice). (B) Intracellular staining of H3K4me3, H3K4me1, or H3K27ac of LSK cells compared with the signal in control IgG (n = 5 or 6 mice) was analyzed by MFI on flow cytometry. (C) JARID demethylase activity and H3K4 methyltransferase activity were measured in Lin^−^ HSPCs (n = 3 or 4 mice). (D) Intracellular staining of HIF-1α of LSK cells was analyzed by MFI on flow cytometry (n = 5 or 6 mice). For all panels, data are shown as the mean ± SEM. *P* values were determined using Student’s *t* test. *P < 0.05; **P < 0.01; n.s., not significant.

Oxidative stress promotes myelopoiesis of HSPCs^46^ and HFD-induced obesity oxidative stress pathway in HSPCs.^11^ Indeed, LSK-HSPCs freshly isolated from HFD mice had increased oxidative stress measured by CellROX indicator dye (Fig. 2A). Epigenetic marks including H3K4me3, H3K4me1, and H3K27ac can program HSPCs to produce myeloid cells with proinflammatory and nonresolution phenotype.^14^ We measured protein levels of H3K4me3, H3K4me1, and H3K27ac and found that H3K4me3, but not H3K4me1 and H3K27ac, was significantly higher in HSPCs isolated from HFD mice compared with those from control LFD mice (Fig. 2B). Next, we found that H3K4me3 demethylase activity was decreased, while H3K4 methyltransferase activity was unchanged in the progenitor-enriched population from HFD mice (Fig. 2C). When we screened demethylases and methyltransferases in HSPCs, we did not find changes in protein levels of KDM5A (JARID1A), Set7/9, and Ash1 using flow cytometry (Fig. S1A, we did not get significant signals with KDM5B and MLL1 antibodies). H3K4me3 demethylases such as KDM5A contain a common prolyl hydroxylase domain **(**PHD) like HIF-1α, and its hydroxylase activity promotes demethylase enzymic activity and HIF-1α degradation. Indeed, there was an increase in HIF-1α levels in HFD-LSK. As oxidative stress can inhibit PHD, metabolic conditions including elevated oxidative stress may contribute to increased H3K4me3 levels in HSPCs in HFD mice. HIF-1α but not H3K4me3 level was increased in myeloid progenitors, and H3K4me3 but not HIF-1α was increased in blood monocytes (Fig. S1B), suggesting that induced H3K4me3 is preserved in a subset of myeloid cells after differentiation of HSPCs like as shown previously.^15^

### CsA reduces oxidative stress and H3K4me3 in HSPCs induced by HFD-induced obesity

**Figure 3.**
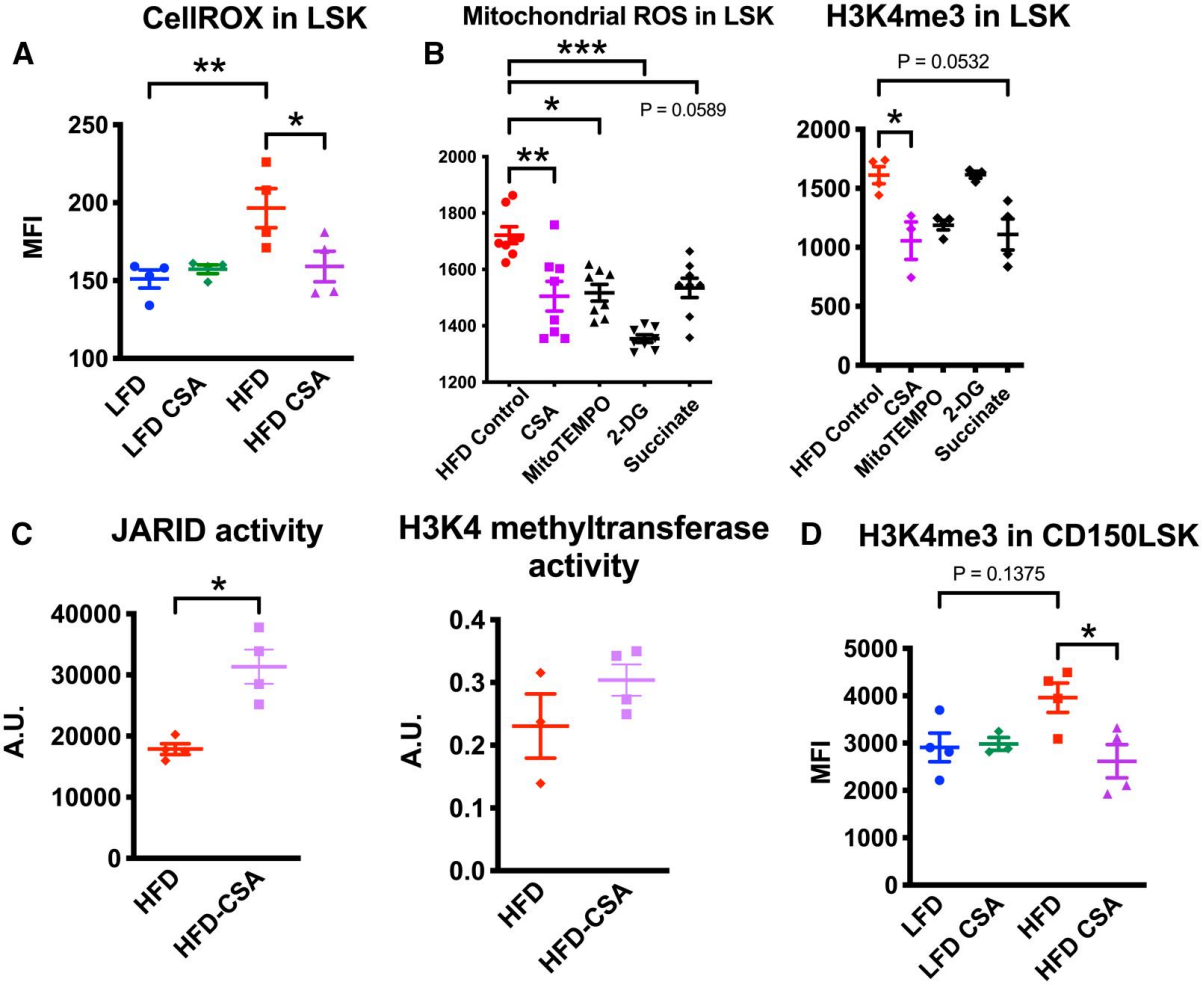
Cyclosporine treatment ex vivo reverses HSPC signatures in HFD-induced obesity. Bone marrow cells from LFD or HFD mice were treated with 50 μg/mL cyclosporine A (CsA) or equivalent volume of dimethyl sulfoxide immediately after BM harvest. (A) Flow cytometry on Lin^−^Sca1^+^cKit^+^ (LSK) BM cells from LFD or HFD mice shows median fluorescent intensity (MFI) of CellRox deep red signals compared with unstained control (n = 4 mice). (B) mitochondrial reactive oxygen species (ROS) and intracellular H3K4me3 in LSK from HFD mice were measured by flow cytometry. MitoTEMPO (mitochondrial ROS scavenger), 2-deoxyglucose or dimethyl succinate was added to the suspension of the cells. (C) the extracted nucleus of Lin^−^ HSPCs from HFD mice was analyzed for the enzymic activity of JARID or KDM5 H3K4 demethylase or H3K4 methyltransferase. (D) intracellular H3K4me3 in CD150^+^LSK from HFD mice were measured by flow cytometry. For all panels, data are shown as the mean ± SEM. Statistical significance was determined using 1-way analysis of variance with Turkey’s multiple comparison test in panels A, B, and D; and using Student’s *t* test in panel C. *P < 0.05; **P < 0.01; *** *P* < 0.001. n.s., not significant.

Innate immune memory associated with H3K4me3 is complex molecular events involving metabolic alteration and cell stimulation likely involving calcineurin.^47^ CsA is a potent calcineurin pathway inhibitor and can improve the long-term function of HS(P)Cs.^23^ CsA treatment indeed reduced overall oxidative stress (Fig. 3A), mitochondrial reactive oxygen species (ROS) and H3K4me3 levels in LSK cells from HFD mice (Fig. 3B). A more specific mitochondrial ROS scavenger, mitoTEMPO, also reduced both mitochondrial ROS and H3K4me3 (Fig. 3B). Interestingly, the glycolysis inhibitor, 2-DG, reduced mitochondrial ROS but not H3K4me3 (Fig. 3B). This suggests that 2-DG modified metabolism is unlikely a driver for elevated H3K4me3 in HSPCs from HFD mice and that H3K4me3 dysregulation is imprinted by HFD and oxidative stress for a longer term in vivo. Succinate addition, which can increase ROS by reverse electron transport at mitochondria’s complex I and inhibit PHD and thus can reduce KDM5 demethylase, showed paradoxical effects (Fig. 3B), suggesting an involvement of an alternative pathway such as succinate-induced flavin adenine dinucleotide and lysine-specific demethylase 1^48^ to maintain H3K4me3. The effect of CsA and other metabolic manipulations did not appear to change H3K4me1 and HIF-1α levels (Fig. S2A, B), and reduction of H3K4me3 by CsA and mitoTEMPO was not observed in LFD-LSK (Fig. S2B). We further found that ex vivo CsA treatment increased H3K4me3 demethylase (JARID) activity but did not affect H3K4 methyltransferase activities (Fig. 3C). We confirmed the reduction of H3K4me3 is specific to HFD in more primitive CD150^+^LSK (Fig. 3D). Thus, CsA treatment inhibits oxidative stress and H3K4me3 in HSPCs induced by HFD-induced obesity.

### CUT&Tag identified differential H3K4me3 loci during myelopoiesis of HSPCs

**Figure 4.**
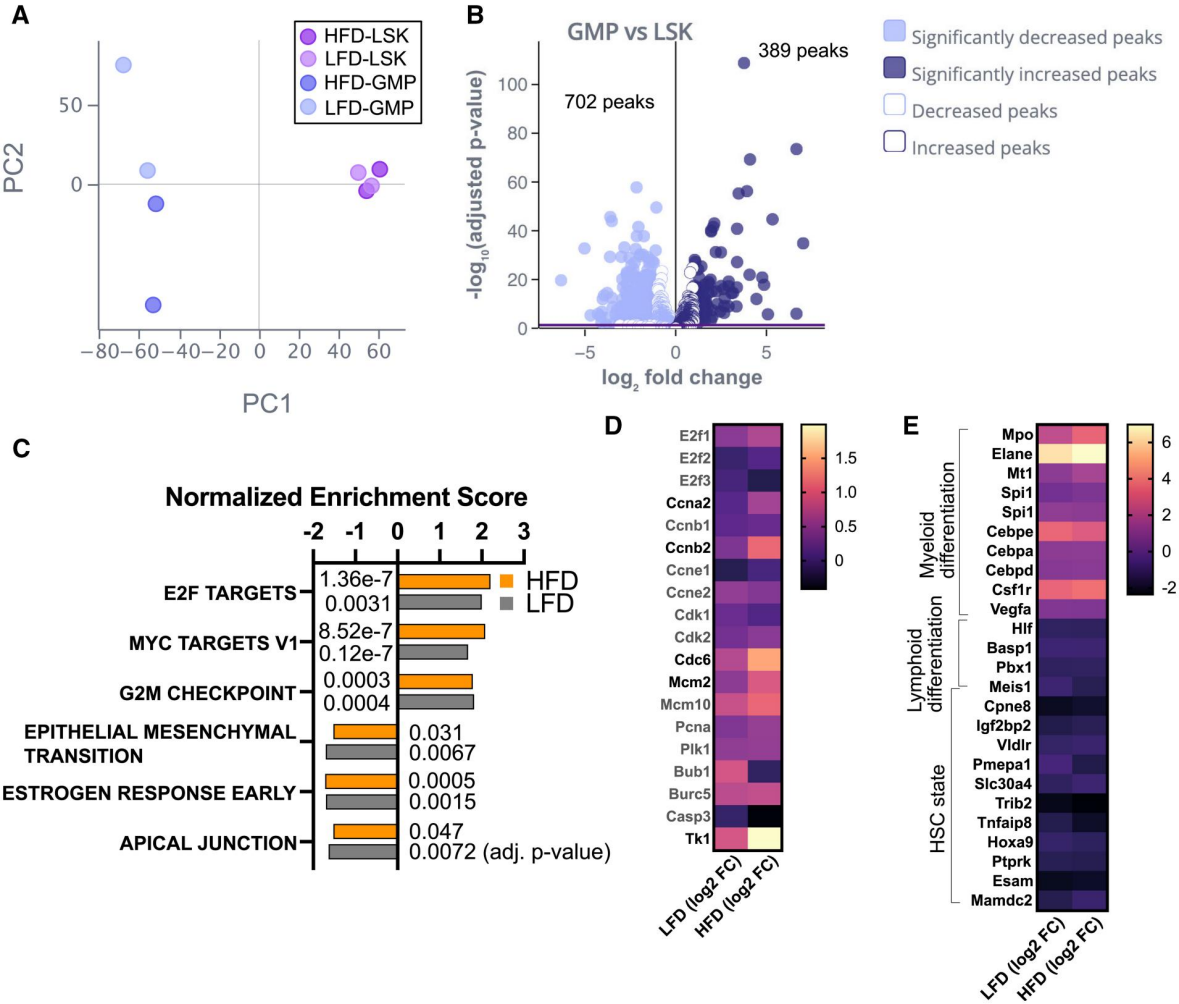
Differential H3K4me3 marks during myelopoiesis. LSK and Lin^−^Sca1^−^cKit^+^FcγR^+^ GMP BM cells from LFD or HFD mice were subjected to CUT&Tag analysis to map H3K4me3 marked genomic locations. (A) Principal component (PC) analysis plot of LSK and GMP from LFD or HFD mice (n = 2 each, GSE291196). (B) Volcano plots of GMP upregulated and downregulated differential H3K4me3 marks compared with LSK (adjusted *P* < 0.01 in solid marks). (C) gene set enrichment analysis of differential H3K4me3 marks (GMP upregulated or downregulated, adjusted *P* values are shown at the side of each bar for normalized enrichment score). (D) Log2 FC of H3K4me3 in representative E2F targets in Hallmark in the transition from LSK to GMP. (E) Lineage signature genes with known chromatin accessibility (ATAC signal) change during myeloid commitment (ups in myeloid differentiation genes and downs in lymphoid differentiation and HSC state genes) curated by literature (see Materials and Methods). Log2 FC of H3K4me3 in lineage signature genes in the transition from LSK to GMP is shown in the heat map.

We next identified the genomic locations that are differently marked by H3K4me3 between LSK and myeloid-committed progenitor cells, GMP cells. Due to the limited abundance of LSK cells in mice, we optimized the CUT&Tag assay in sorted HSPCs and found 94.1% of consensus peaks within ±1,000 kb of transcription start site of gene promoters. In a small cohort (n = 2 for each condition/population), we saw significant differential H3K4me3 peaks between LSK and GMP and much less between LFD and HFD mice in each cell population (Fig. 4A). We identified 389 upregulated and 702 downregulated H3K4me3 during the transition from LSK to GMP (adjusted *P* < 0.01, fold change [FC] > 2 or FC < 0.5) (Fig. 4B; Tables S1 and S2). GSEA showed E2F targets, G2M checkpoint, Myc targets, and oxidative phosphorylation in the upregulated H3K4me3, and estrogen response early, apical junction, and epithelial-mesenchymal transition in the downregulated H3K4me3 in both LFD and HFD mice (Fig. 4C), with a tendency of higher significance in E2F and Myc targets in HFD mice. E2F transcription factors are required for the activation of genes involved in DNA synthesis and cell-cycle progression, and activation of these E2F targets is mediated by inhibition of the retinoblastoma (Rb) transcription factor and its binding KDM5A H3K4me3 demethylase.^49^ Consistent with the inhibited KDM5 (JARID) demethylase activity in HFD mice (Fig. 2C), we found increases of H3K4me3 from LSK to GMP transition in individual E2F targets such as Ccnb2, Cdc6, Mcm2, and Tk1 in HFD mice (Fig. 4D).

Using available data from ATAC sequencing in HSPCs,^10^^,^^39–42^ we curated a list of genes that show opening or closing chromatin based on increase/decrease of ATAC signal during myelopoiesis. Overlapping of H3K4me3 upregulation during the transition from LSK to GMP with chromatin opening during myelopoiesis was small (9/168 chromatin opening), and overlapping of H3K4me3 downregulation during the transition from LSK to GMP with chromatin closing during myelopoiesis is also small (15/129 chromatin closing) (Fig. S3A). Mpo, Elane encoding neutrophil elastase, Spi1 encoding PU.1, Cebpd, Cebpe, Cebpa, Csf1r, and Vegfa, which are signature myeloid populations, were significantly upregulated in both the H3K4me3 level and ATAC signal, while a negatively enriched ATAC signal in lymphoid differentiation and HSC state was associated with downregulation of H3K4me3 (Fig. 4E; Fig. S3B). Differential H3K4me3 loci detected by our CUT&Tag capture key epigenetic events during the transition from LSK to GMP, which highlights the upregulation of H3K4me3 at key myelopoietic signature genes. The E2F targets as well as Myc targets, for which gene expression is increased in HSPC activation/proliferation but decreased during myeloid specification and differentiation,^39^^,^^50^ have enriched H3K4me3 in HFD in transition from LSK to GMP. This may reflect the inhibition of KDM5A demethylase during myeloid differentiation in HFD as KDM5A binding Rb protein regulating E2F,^51^ regulating cell cycling, differentiation,^52^ and mitochondria function.^49^^,^^53^

### CUT&Tag identified H3K4me3 upregulated loci in HSPCs from HFD mice

**Figure 5.**
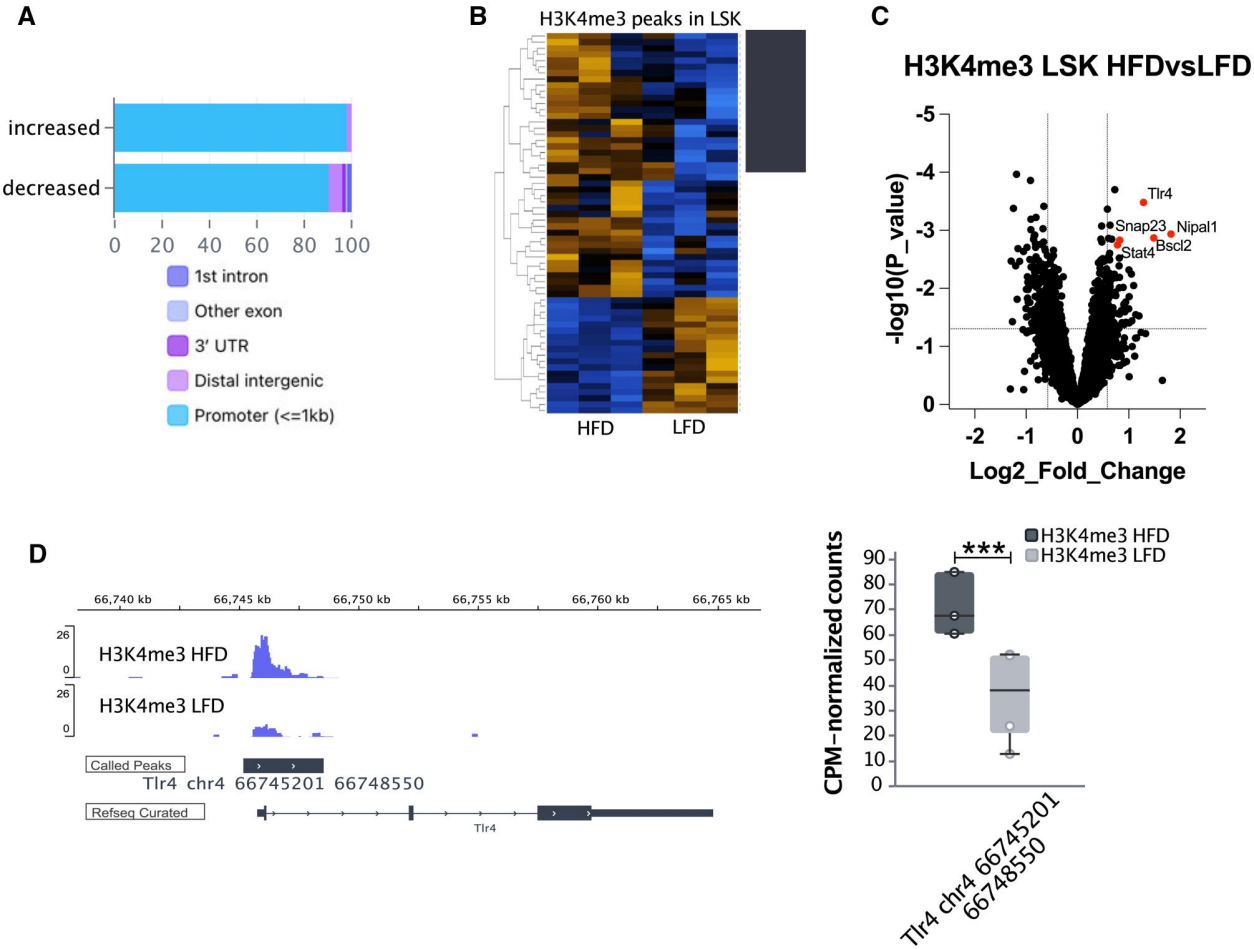
Differential H3K4me3 marks between LFD-LSK and HFD-LSK. LSK cells from LFD and HFD mice were subjected to CUT&Tag for H3K4me3 (n = 3, GSE256500). (A) Peak annotations of 14,659 consensus peaks in all 7 samples. (B) Clustering analysis was performed for H3K4me3 using differentially abundant features in the comparison of HFD vs LFD. Features were filtered using an adjusted *P* value ≤0.01 and log2 FC threshold of 1 (showing features that are both positive and negative). Heatmap shows counts per million (CPM)–normalized, log2-transformed, and z score–transformed values. Samples are colored by diet. Dendrograms show clustering by Euclidean distance for targets. (C) Volcano plots of HFD-LSK upregulated and downregulated differential H3K4me3 marks compared with LFD-LSK (unadjusted *P* < 0.05; FC > 1.5 or <0.75 were shown by dotted lines). (D) Peaks are visualized on an Integrative Genome Viewer at indicated genomic locations. Boxplot showing the CPM-normalized values for *Tlr4* promoter lesions. Boxplots are drawn using the median to define interquartile ranges, with outliers defined as ±1.5 × the interquartile range from the third quartile or first quartile, respectively. *** *P* ≤ 0.001.

We next identified the genomic locations that are differently marked by H3K4me3 between LSK cells from HFD mice and those from LFD mice as an indication of obesity-induced epigenetic change. Because of the small difference between LFD and HFD in LSK in the first cohort (Fig 4A), we added another cohort to identify the effect of diets on differential H3K4me3 loci in LSK. We identified a total of 14,659 H3K4me3 peaks, which were located predominantly at promoter regions. The differential peak analysis revealed 124 significantly increased (FC > 1.5, *P* < 0.01) and 99 decreased (FC < 0.75, *P* < 0.01) H3K4me3 peaks in HFD-LSK, predominantly at gene promoters (Fig. 5A, B; Table S3). Running GSEA, only apical junction in Hallmark gene sets, which was downregulated during the transition from LSK to GMP (Fig. 4C), was coincidently decreased in H3K4me3 of HFD-LSK (adjusted *P* = 0.02). We found *Tlr4*, *Nipal1*, and *Bscl2* in the top of the significantly increased H3K4me3 in HFD-LSK (Fig. 5C, D). When we analyzed H3K4me3 CUT&Tag data in GMP, we only found 8 gene peaks (*Tspan14*, *Gcnt2*, *Bscl2*, *Ctso*, *Zfp939*, *Cdc73*, Sec11a, *Napb*) that commonly upregulated in LSK and GMP from HFD (Fig. S4, Table S4), suggesting that differential H3K4me3 marks are highly cell lineage specific^54^^,^^55^ or may be masked by heterogeneity within LSK and GMP.

### TLR4-mediated myelopoiesis is enhanced in HSPCs from HFD mice

**Figure 6.**
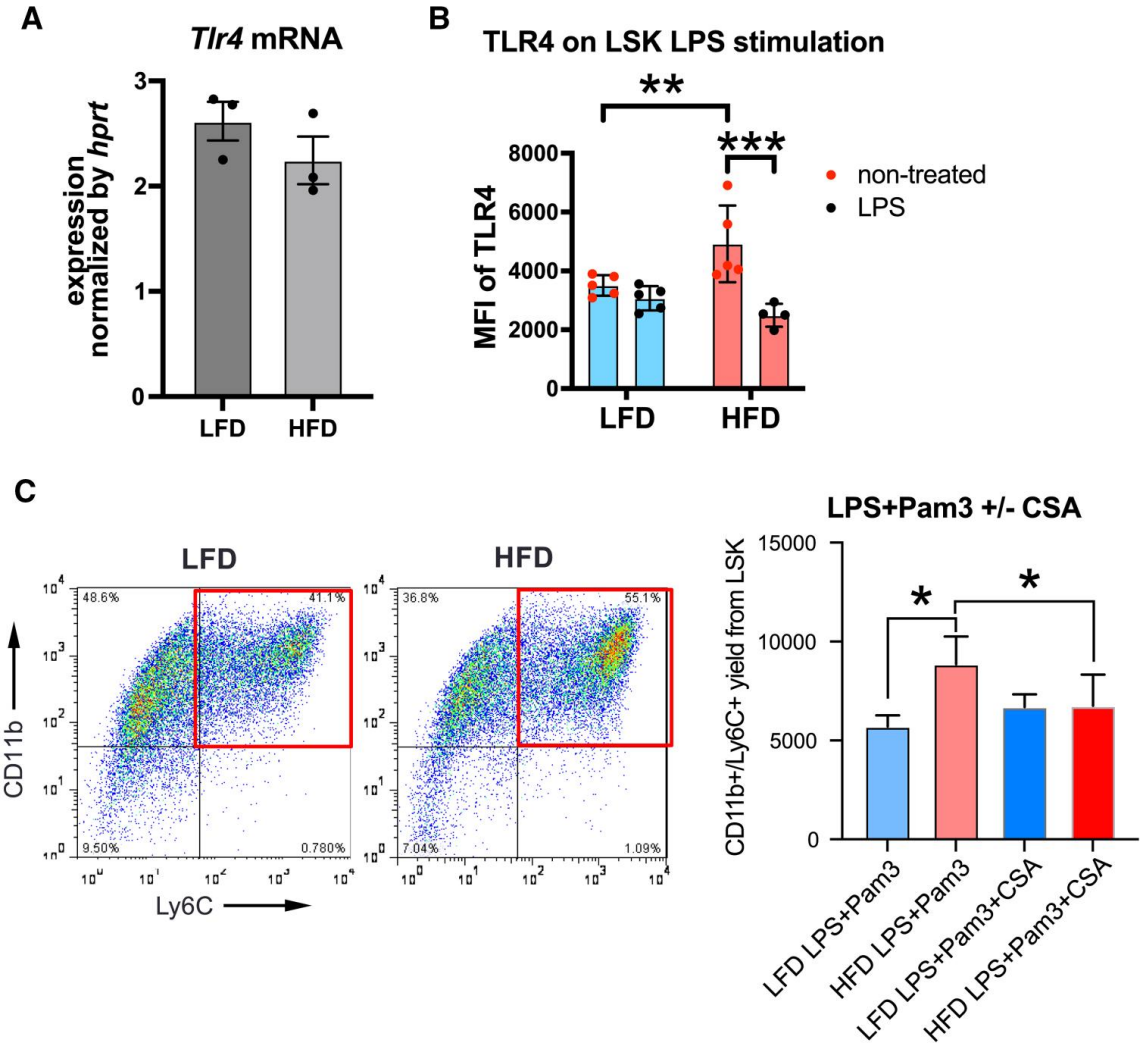
Increased TLR4 responsiveness and its induced myelopoiesis in LSK cells from HFD mice. (A) Quantitative PCR analysis of *Tlr4* gene expression in sorted LSK cells from LFD or HFD mice (n = 3). (B) TLR4 surface expression under cell culture conditions was measured by flow cytometry in LSK between LPS (100 ng/mL) treated and nontreated conditions (n = 4 or 5 mice). (C) Sorted LSK cells from LFD and HFD mice were cultured in StemPro34 media supplemented with SCF (20 μg/mL) and Flt3-L (20 μg/mL) and stimulated with LPS (1 μg/mL) and Pam3CSK4 (100 ng/mL) for 72 h, with/without CsA (50 μg/mL) treatment. CD11b^+^/Ly6C^+^ myeloid cells were quantified by cell counting and flow cytometry (n = 3–6 biological replicates). For all panels, data are shown as the mean ± SEM. Statistical significance was determined using 1-way analysis of variance with Turkey’s multiple comparison test in panels B and C; using Student’s t test in panel A. *P < 0.05; **P < 0.01; *** *P* < 0.001. mRNA, messenger RNA.

To determine whether upregulated H3K4me3 at the TLR4 gene promoter leads to increased TLR4 levels in LSK, we performed reverse-transcriptase PCR of sorted LSK cells and found no difference of Tlr4 gene expression (Fig. 6A). However, when we analyzed surface expression of TLR4 on HSPCs using flow cytometry, we found increased TLR4 on cell surface of LSK cells from HFD mice compared with those from LFD mice under cell culture condition. stimulation with a TLR4 ligand, LPS reduced TLR4 levels, indicating that TLR4 can be internalized and is functional (Fig. 6B). TLR4 stimulation in LSK cells induces myelopoiesis, particularly their differentiation into monocyte lineage,^56^ and this effect is amplified with costimulation by the TLR2 ligands, Pam3CK4.^57^ LSK cells from HFD mice showed an enhanced response to TLR2/4 ligands, generating higher numbers of monocytes/macrophages compared with LFD-HSPCs (Fig. 6C). In addition, CsA treatment, which reduces HFD-induced elevation of H3K4me3 levels (Fig. 3A, D), was sufficient to reduce myelopoiesis of LSK cells from HFD mice in culture.

**Figure 7.**
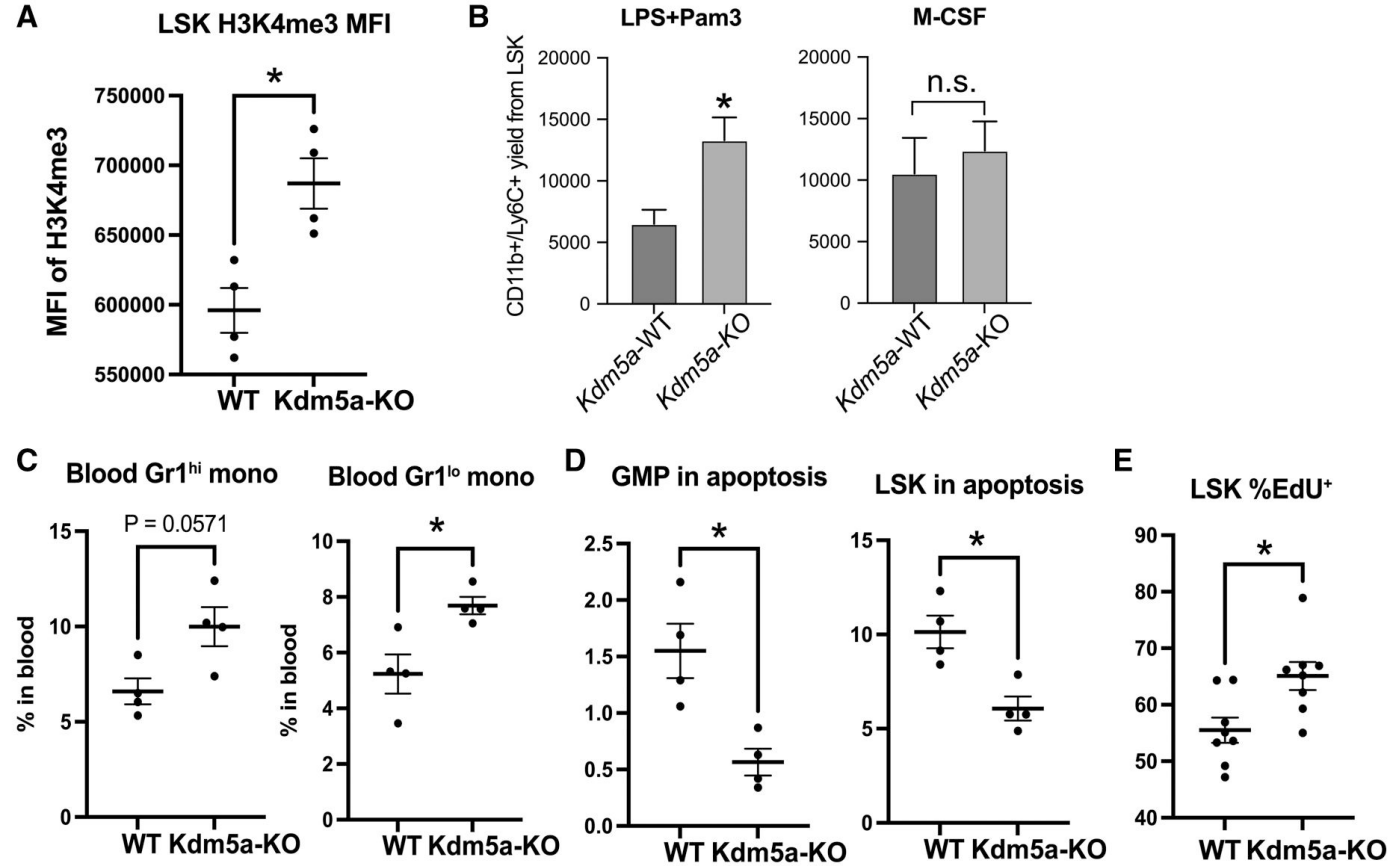
Increased H3K4me3 by KDM5A KO enhances myelopoiesis bias. Kdm5a demethylase KO was achieved by tamoxifen (Tam)-induced Cre/lox system. Tam injected Cre− was served as control wild-type (WT). (A) Increased intracellular H3K4me3 levels in LSK BM cells from Kdm5a-KO was shown by median fluorescence intensity (MFI) in gated LSK cells in flow cytometry analysis (n = 4 mice). (B) Sorted LSK cells from WT or Kdm5a-KO mice were cultured in StemPro34 media supplemented with SCF (20 μg/mL) and stimulated with LPS (1 μg/mL) and Pam3CSK4 (100 ng/mL) for 72 h. CD11b^+^/Ly6C^+^ myeloid cells were quantified by cell counting and flow cytometry (culture triplicates of n = 3 mice). (C) Flow cytometry analysis of the blood for CD115^+^/Gr1^hi^ and CD115^+^/Gr1^lo^ monocytes in total leukocytes (n = 4 mice). (D) Annexin V+ fractions of live-cell gated Lin^−^Sca1^−^cKit^+^FcγR^+^ GMP and LSK BM cells (n = 4 mice) were analyzed by flow cytometry. (E) EdU (600 μg per mouse) was injected 4 h before harvesting BM cells. EdU^+^ fraction of LSK BM cells (n = 8 mice) was analyzed by flow cytometry. For all panels, data are shown as the mean ± SEM. Statistical significance and *P* value were determined using Student’s *t* test. *P < 0.05. n.s., not significant.

Because our findings indicated that oxidative stress induced by HFD-induced obesity inhibits KDM5 demethylase, we performed experiments using KDM5a demethylase–inducible KO mice. We confirmed tamoxifen-inducible KO at the protein level in multiple organs/tissues (Fig. S5A). KO of KDM5a resulted in upregulation of H3K4me3 levels in HFD-LSK (Fig. 7A) and enhanced TLR2/4 ligand–induced myelopoiesis of LSK cells. In contrast, myelopoiesis by M-CSF was not affected by KDM5a KO (Fig. 7B). In vivo, KDM5a KO increased blood monocyte levels (Fig. 7C) but did not alter myeloid progenitor levels in the BM (data not shown). The rate of apoptosis was decreased in GMP and LSK cells from KDM5A-KO mice (Fig. 7D), and cell proliferation was increased in LSK cells from KDM5A-KO mice (Fig. 7E), consistent with the phenotype found in KDM5A global KO.^52^ These data support the notion that inhibition of KDM5A by oxidative stress may causally contribute to increased TLR4 responsiveness and myelopoiesis-promoted phenotype in HFD-induced obesity.

We next determined whether KDM5A-KO further enhances its myelopoietic phenotype in response to HFD-induced obesity. The gene KO was introduced after developing obesity by 12 wk of HFD feeding in B6 mice. This acute KDM5A-KO (2–3 wk after the final tamoxifen injection) did not affect body weight or blood glucose, and did not further expand myeloid compartments (not shown) or further increase TLR4 expressions in LSK (Fig. S5B). KDM5A-KO was not sufficient to further increase myelopoietic phenotype induced by HFD-induced obesity. However, when we challenged HFD-fed KDM5A-KO with dorsal skin biopsy injury, higher levels of monocytes in spleen and neutrophils in blood and spleen were observed compared with KDM5A-intact HFD mice (Fig. S5C, D), indicating promotion of the myelopoietic phenotype. These suggest that low activity of KDM5A is linked with enhanced myeloid output in response to tissue injury in the HFD-induced obesity environment.

## CsA treatment reverses dysregulated HSPCs by HFD-induced obesity

**Figure 8.**
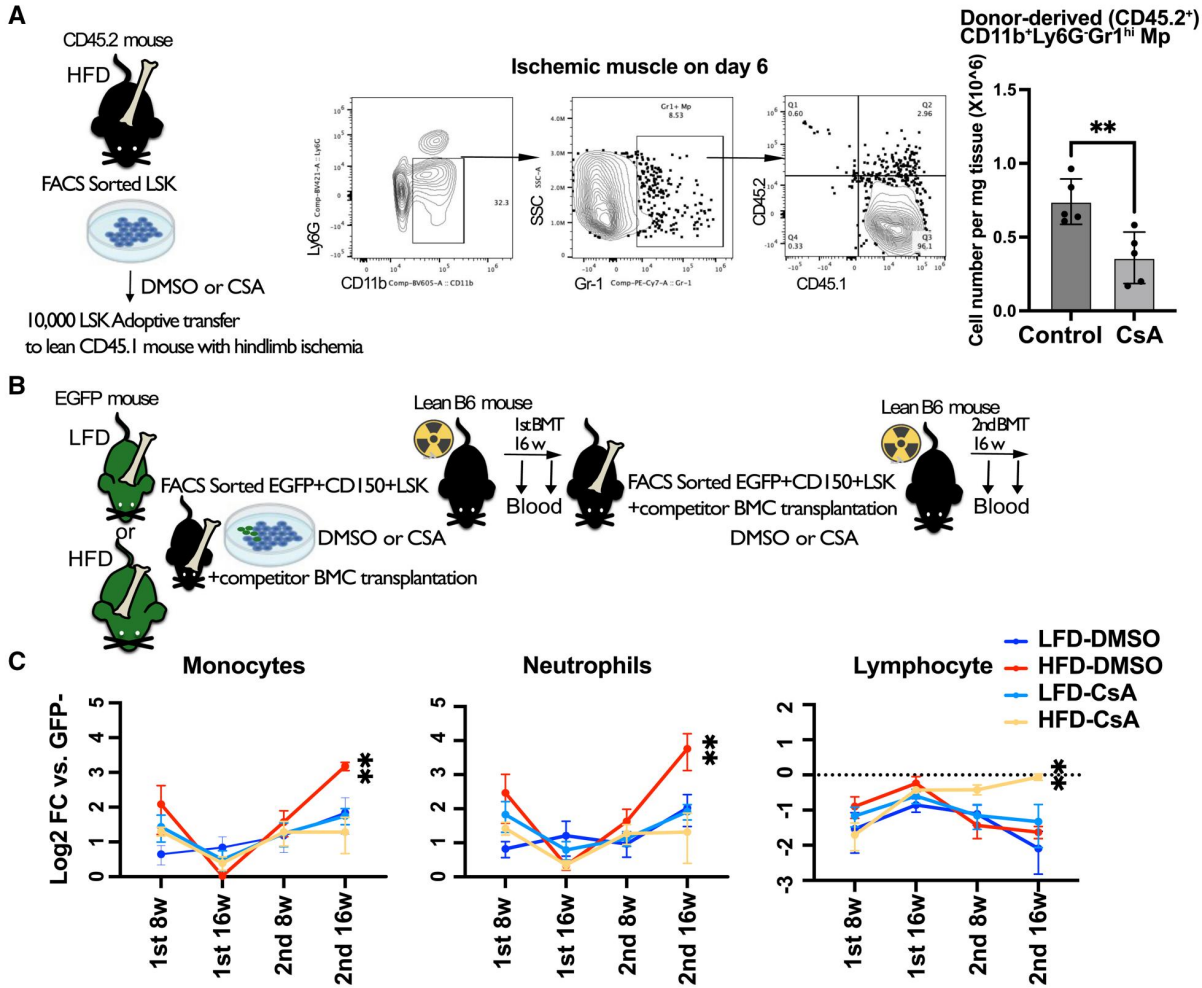
Obesity induces long-term nonresolution phenotype in macrophages, which can be reversed by ex vivo CsA treatment on HSPCs. (A) LSK cells from HFD mice were ex vivo treated with CSA (50 μg/mL) and were intravenously injected to lean CD45.1 mice just after hindlimb ischemia surgery. After 6 d, the ischemic tibialis anterior were analyzed. Donor-derived CD45.2^+^/CD11b^+^/Ly6G^−^ monocytes/macrophages were quantified (n = 5 mice). (B) A scheme for serial competitive BM transplantation assay. EGFP^+^ CD150^+^LSK cells sorted from LFD- and HFD-fed EGFP mice were ex vivo treated with CSA (50 μg/mL) and were intravenously injected to lean (EGFP^−^) mice lethally irradiated (9.5 Gy) along with (EGFP^−^) competitor BM cells. Peripheral blood was collected 8 and 16 wk after transplantation, and a second transplantation was performed. BMDMs were grown from the BM cells isolated 16 wk after the second transplantation. (C) Flow cytometry analysis of the blood at indicated time points shows donor-derived (EGFP^+^) cells by a relative FC compared with EGFP^−^ cells in each cell population (neutrophils: Ly6G^+^CD11b^+^; monocytes: Ly6G^−^CD11b^+^; lymphocytes: Ly6G^−^CD11b^−^) (n = 3–6 mice). For all panels, data are shown as the mean ± SEM. Statistical significance was determined using Student’s *t* test in panel A; using 2-way analysis of variance with Turkey’s multiple comparison test in panel B. **P < 0.01.

Finally, we determined whether CsA treatment can reverse proinflammatory myelopoietic programming of HSPCs induced by HFD-induced obesity. LSK cells from HFD mice were treated or not with CsA ex vivo and then intravenously injected into lean recipients immediately after hindlimb ischemia. We found that CsA treatment reduced Gr-1^hi^ (inflammatory) monocytes/macrophages derived from the donor LSK cells in ischemic muscle (Fig. 8A). To study long-term myelopoiesis bias in HSPCs, we performed serial competitive BM transplantation in sublethally irradiated recipients using EGFP mice as HSPC donor sources from either LFD or HFD mice, using CD45.1 BM cell competitor. We did not find significant differences in reconstitution ability among the groups. However, we found increased myeloid bias in HFD-HSPCs after the second BM transplantation, consistent with a previous report,^59^ and that CsA treatment upon each BM transplantation successfully reversed the HFD-imprinted proinflammatory myelopoiesis bias in HSPCs (Fig. 8B).

By linking oxidative stress to epigenetic modifications in HSPCs, we identify a potential therapeutic target to reverse obesity-induced inflammation. Given the growing burden of obesity-related inflammatory diseases, further investigation into interventions that reprogram hematopoietic memory may have broad clinical implications.

## Discussion

The long-term impact of obesity on hematopoiesis and immune function remains incompletely understood, particularly in the context of tissue injury and repair. Here, we demonstrate that HSPCs from HFD-induced obese mice undergo persistent epigenetic and functional alterations that promote inflammation and impair tissue recovery. Using BM transplantation models, we show that HFD-induced cell-intrinsic changes in HSPCs are sufficient to impair tissue repair in lean recipient mice following ischemic injury through proinflammatory myelopoiesis bias. We identified oxidative stress in HSPCs is linked with increased H3K4me3 levels along with reduced KDM5 demethylase activity, resulting in increased responsiveness to TLR4 and proinflammatory myelopoiesis. Newly discovered H3K4me3 enrichments at the TLR4 promoter and E2F targets highlight epigenetic imprinting by diet-induced obesity within HSPCs. Thus, our findings further indicate that innate immune programming can originate from HSPCs and be passed down to innate immune cells through myelopoiesis.^7^^,^^9^^,^^10^^,^^14^

Mechanistically, we identified oxidative stress as a key driver of HSPC dysfunction in obesity. Elevated ROS in HFD-HSPCs led to increased trimethylation of histone H3K4, a modification linked to transcriptional activation and innate immune memory.^10^^,^^47^^,^^59^ While increased H3K4me3 appears to be mediated by reduced H3K4me3 demethylase (KDM5) activity in our study (Fig. 2C), other studies have implicated MLL1 methyltransferase in increasing H3K4me3 levels at GMP levels of HFD mice.^15^ As these histone methylation modifying enzymes are regulated by redox state, metabolites and oxygen availability,^59^ changes in the HSPC microenvironment likely drive long-term alteration of HSPCs.

Epigenomic profiling using CUT&Tag further revealed significant H3K4me3 changes at loci associated with myeloid differentiation and inflammatory signaling. In particular, we identified increased H3K4me3 at the *Tlr4* promoter in HFD-HSPCs, accompanied by heightened surface protein expression of TLR4 and enhanced myelopoiesis upon TLR2/4 ligand stimulation. A previous study identified HFD-induced chromatin opening^10^ and H3K4me3 at *Tlr4* loci^15^ in myeloid progenitor cells. A TLR4 ligand, LPS can alter HSPCs’ long-term fitness through oxidative stress,^60^ and LPS-induced TLR4 activation was sufficient to recapitulate myeloid-biased phenotype in HFD mice.^61^ LPS-induced epigenetic changes in HSPCs are partly explained by chromatin opening at C/EBPβ binding enhancer regions rather than promoters.^42^ Other ligands for TLR4 likely contribute to persistent reprogramming of myeloid cells, as shown by unsaturated fatty acid in diet-induced obesity.^5^ Consequently, the heightened TLR4 responsiveness of HFD-HSPCs amplifies LPS- or TLR-driven epigenetic reprogramming, potentially leading to maladaptive HSPC programming^60^ and reinforcing the persistence of proinflammatory myelopoiesis.

We also found H3K4me3 enrichment at E2F targets and myeloid signature genes (such as genes encoding myeloperoxidase, neutrophil elastase, PU.1, Cebpd, Cebpe, Cebpa, and Csf1r) during normal myeloid differentiation of HSPCs (Fig. 4). KDM5A modifies E2F targets through H3K4me3 and Rb,^352^^,^^53^^,^^62^ and along with its homolog KDM5B, is relatively active in normal undifferentiated and nonproliferative HSPCs.^63^ Our data suggest H3K4me3 enrichment at E2F targets and Myc targets during myelopoiesis are augmented in HFD (Fig. 4). Increased H3K4me3 via inactivated KDM5A likely contributes to enhanced myeloid differentiation and increased cell cycling, consistent with our data using KDM5A-KO (Fig. 7), and with the previous studies using KDM5A global KO^52^ or Rb KO in HSPCs.^64^ Fine-tuned RB-E2F regulation in an intermediate state of its activity seems to be crucial for cell cycle progression.^65^ Increased cMyc in HSCs in HFD mice^11^^,^^58^ may also contribute to H3K4me3 enrichment in Myc targets during myelopoiesis in HFD. E2F and Myc targets are normally inhibited during differentiation from multipotential progenitor to GMP (based on bulk RNA sequencing data GSE162607)^39^ and are potentiated in LPS-induced epigenetic memory in HSPCs.^42^ Increased enrichment of H3K4me3 during LSK to GMP transition in HFD may represent transcriptional memory during myelopoiesis, likely reflecting higher E2F and Myc activities due to KDM5 inhibition and high sensitivity to LPS in LSK cells. H3K4me3 is a hallmark of active promoters, but its precise role in transcription remains debated.^59^ Beyond transcriptional initiation, it facilitates RNA polymerase II pause-release for productive elongation.^66^ H3K4me3 also counteracts Polycomb group proteins (H3K27me3)^67^ and DNA methylation.^54^^,^^68^ Its role at Tlr4 and other loci like E2F and Myc in myelopoiesis remains to be clarified.

To investigate therapies for reversing obesity-induced inflammation, we tested CsA, an inhibitor of calcineurin that is known to transmit signals from beta-glucan, which is implicated in innate immune memory.^47^ CsA reduced oxidative stress^23^ and likely inflammatory gene expression^69^ in HSPCs by inhibiting the mitochondrial permeability transition pore.^23^ Our data confirm that CsA lowered oxidative stress and normalized H3K4me3 levels in HFD-HSPCs, reducing their heightened TLR4 responsiveness. While mitoTEMPO also lowered H3K4me3, glycolysis inhibition via 2-DG did not, indicating that mitochondrial metabolism drives obesity-induced H3K4me3 changes. Since CsA is a potent immunosuppressant inhibiting T cells, systemic CsA treatment should not be considered as a therapeutic approach. However, harnessing HSPC-specific CsA pathways could offer a more precise approach to restoring hematopoietic homeostasis. Similarly, inhibiting the Ca^2+^/calcineurin pathway in Ob/Ob mice with Tet2^–/–^ cells reversed their myelopoietic phenotype.^70^ These findings highlight how obesity reprograms hematopoiesis to sustain inflammation and impair tissue repair, suggesting metabolic and epigenetic targeting in HSPCs as a potential therapeutic strategy.

## Supplementary Material

vkaf156_Supplementary_Data

## Data Availability

CUT&Tag data in this study are available in Gene Expression Omnibus (https://www.ncbi.nlm.nih.gov/geo) with GEO accession numbers GSE256500 for CUT&Tag of LSK cells and GSE291196 for CUT&Tag of LSK and GMP cells. All other relevant data are available from the corresponding authors.
