# Supplementary material for: Diet-induced obesity induces oxidative stress and enhances H3K4me3 levels, driving nonresolving inflammation and myelopoiesis in hematopoietic stem and progenitor cells
Source: J Immunol. 2025 Aug 1;214(10):2715–29. doi: 10.1093/jimmun/vkaf156 (PMC12576131; doi:10.1093/jimmun/vkaf156)
Supplement: vkaf156_Supplementary_Data [file vkaf156_supplementary_data.pdf]

Figure S1

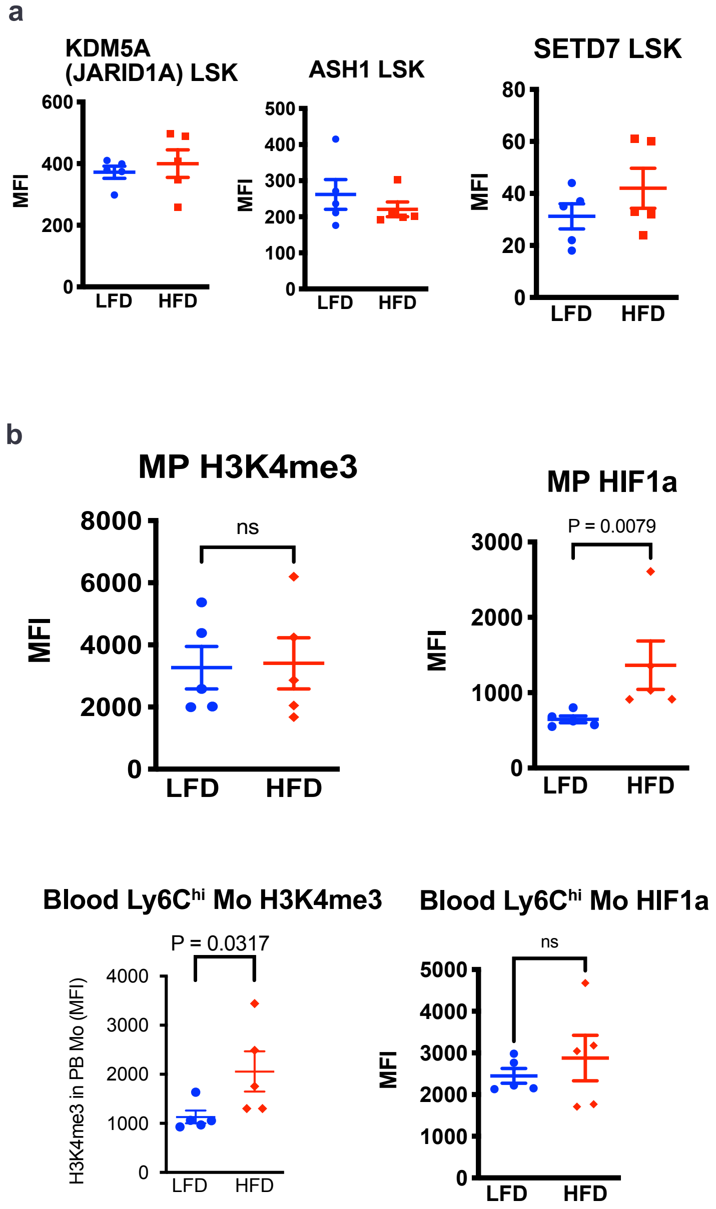

a
KDM5A (JARID1A) LSK
MFI
0
200
400
600
LFD
HFD
ASH1 LSK
MFI
0
100
200
300
400
500
LFD
HFD
SETD7 LSK
MFI
0
20
40
60
80
LFD
HFD
b
MP H3K4me3
MFI
0
2000
4000
6000
8000
ns
LFD
HFD
MP HIF1a
MFI
0
1000
2000
3000
P = 0.0079
LFD
HFD
Blood Ly6Chi Mo H3K4me3
P = 0.0317
H3K4me3 in PB Mo (MFI)
0
1000
2000
3000
4000
LFD
HFD
Blood Ly6Chi Mo HIF1a
ns
MFI
0
1000
2000
3000
4000
5000
LFD
HFD

Supplemental Figure 1: a, flow cytometry analysis of intracellular staining of H3K4 modifying molecules, KDM5A (JARID1A), ASH1 and SETD7 in $Lin^{-}Sca1^{+}cKit^{+}$ (LSK) gated population of bone marrow cells from LFD or HFD mice. The expression levels were compared by median fluorescent intensity (MFI) of the difference between the target stained and IgG (n=5 mice). **b**, flow cytometry analysis of intracellular staining of H3K4me3 or HIF-1α in $Lin^{-}Sca1^{-}cKit^{+}$ myeloid population (MP) gated population of bone marrow cells from LFD or HFD mice (n=5 mice). Statistical significance and P-value were determined using Student's t-test. n.s.; not significant.

Figure S2

a

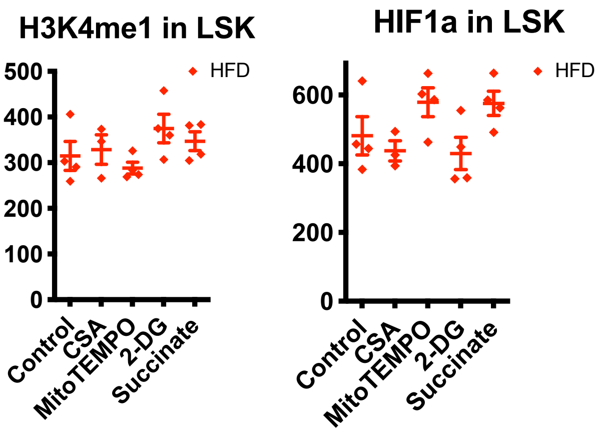

H3K4me1 in LSK
HIF1a in LSK
HFD
500
400
300
200
100
0
600
Control
CSA
MitoTEMPO
2-DG
Succinate

b

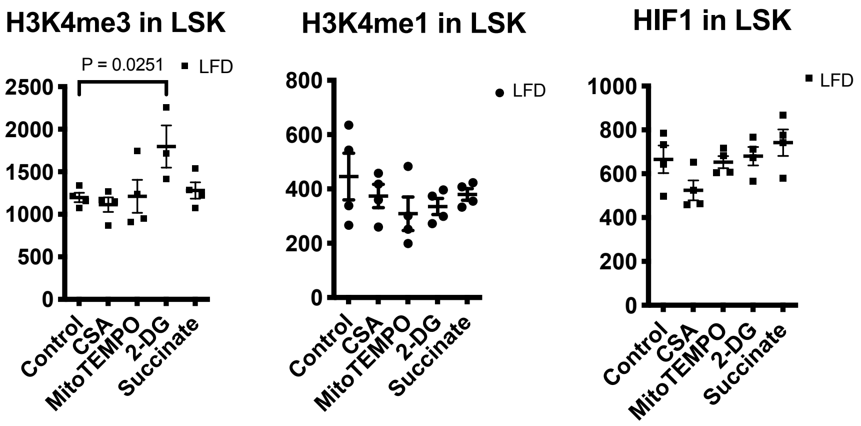

H3K4me3 in LSK
H3K4me1 in LSK
HIF1 in LSK
P = 0.0251
LFD
2500
2000
1500
1000
500
0
800
600
400
200
Control
CSA
MitoTEMPO
2-DG
Succinate

Supplemental Figure 2: Bone marrow cells from LFD or HFD mice were treated with 50 μg/ml cyclosporine A (CsA) or equivalent volume of DMSO immediately after bone marrow harvest. MitoTEMPO (mitochondrial ROS scavenger), 2-deoxyglucose or di-methylsuccinate was added to the suspension of the cells. **a**, flow cytometry analysis of intracellular staining of H3K4me1 or HIF-1α in $Lin^{-}Sca1^{+}cKit^{+}$ (LSK) gated population of bone marrow cells from HFD mice (n=3-5 mice). **b**, flow cytometry analysis of intracellular staining of H3K4me3, H3K4me1 or HIF-1α in $Lin^{-}Sca1^{+}cKit^{+}$ (LSK) gated population of bone marrow cells from LFD mice (n=3-5 mice).

Figure S3

e

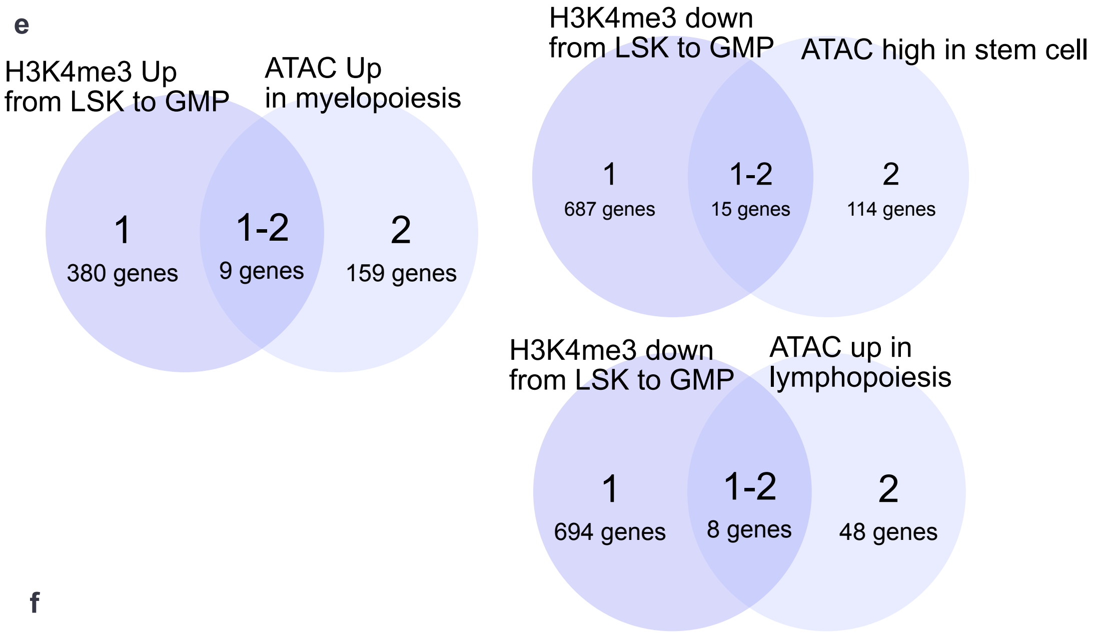

f

**Category**	**Genes**
Myeloid Overlap	Mpo, Elane, Mt1, Spi1, Cebpd, Cebpe, Csf1r, Vegfa, Cebpa
Lymphoid Overlap	Hlf, E2f6, Foxo1, Basp1, Slc30a4, Pbx1, Cobll1, Meis1
Stem Cell Overlap	Cpne8, Igf2bp2, Bach2, Ndrg1, Foxo1, Vldlr, Pmepa1, Slc30a4, Trib2, Tnfaip8, Hoxa9, Ptprk, Meis1, Esam, Mamdc2

Supplemental Figure 3: a, overlapping of H3K4me3 upregulation in GMP compared to LSK (GSE291196) (389 genes) with chromatin opening (ATAC signal upregulation) during myelopoiesis curated from pieces of literature [10,25-28], H3K4me3 downregulation with ATAC signal enriched in hematopoietic stem cells, and H3K4me3 downregulation with ATAC signal enriched in lymphoid progenitors. **b**, list of gene locations that are identified as overlaps between H3K4me3 and ATAC signals. Myeloid overlap indicates gains in both H3K4me3 and ATAC signals during myelopoiesis (GMP vs. LSK), and lymphoid or stem cell overlaps indicate losses in both H3K4me3 and ATAC signals during myelopoiesis (GMP vs. LSK).

Figure S4

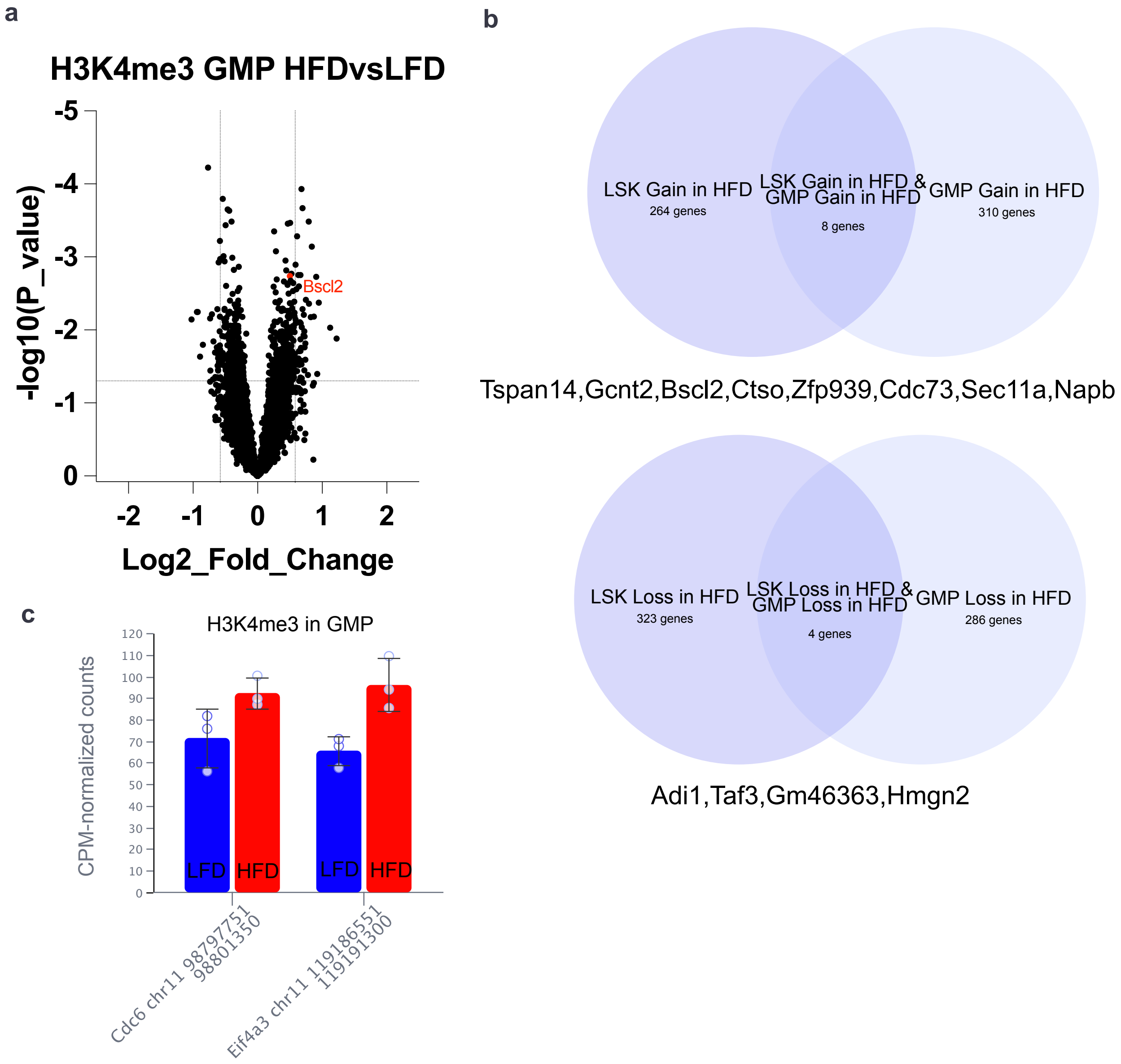

a
H3K4me3 GMP HFDvsLFD
-log10(P_value)
-5
-4
-3
-2
-1
0
Bscl2
-2
-1
0
1
2
Log2_Fold_Change
b
LSK Gain in HFD
264 genes
LSK Gain in HFD & GMP Gain in HFD
8 genes
GMP Gain in HFD
310 genes
Tspan14,Gcnt2,Bscl2,Ctso,Zfp939,Cdc73,Sec11a,Napb
LSK Loss in HFD
323 genes
LSK Loss in HFD & GMP Loss in HFD
4 genes
GMP Loss in HFD
286 genes
Adi1,Taf3,Gm46363,Hmgn2
c
H3K4me3 in GMP
CPM-normalized counts
120
110
100
90
80
70
60
50
40
30
20
10
0
LFD
HFD
LFD
HFD
Cdc6 chr11 98797751 98801350
Eif4a3 chr11 119186551 119191300

Supplemental Figure 4: a, Differential H3K4me3 marks in granulocyte-macrophage progenitor cells (GMP; $Lin^{-}Sca1^{-}cKit^{+}Fc\gamma R^{+}CD34^{+}$) in the bone marrow between LFD and HFD mice (n=3, GSE291196). **b**, the overlaps of the genes associated with H3K4me3 peaks upregulated in HFD in both LSK (Fig. 5c) and GMP (Fig. S3a). **c**, the overlaps of the genes associated with H3K4me3 peaks downregulated in HFD in both LSK (Fig. 5c) and GMP (Fig. S3a).

Figure S5

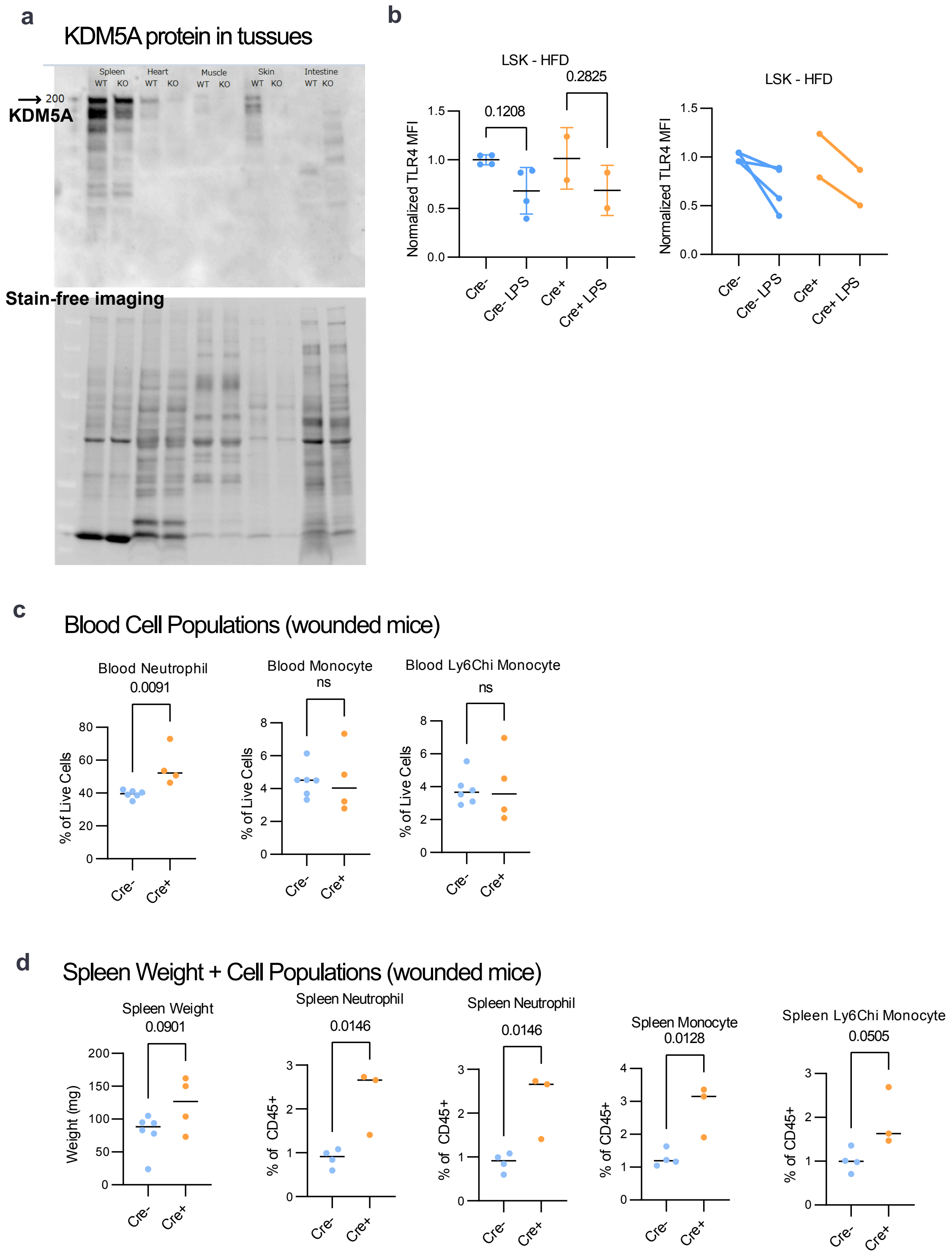
a
KDM5A protein in tussues
Spleen
WT KO
Heart
WT KO
Muscle
WT KO
Skin
WT KO
Intestine
WT KO
200
KDM5A
Stain-free imaging
b
LSK - HFD
0.1208
0.2825
Normalized TLR4 MFI
1.5
1.0
0.5
0.0
Cre-
Cre- LPS
Cre+
Cre+ LPS
LSK - HFD
c
Blood Cell Populations (wounded mice)
Blood Neutrophil
0.0091
% of Live Cells
80
60
40
20
0
Cre-
Cre+
Blood Monocyte
ns
8
6
4
2
Blood Ly6Chi Monocyte
ns
d
Spleen Weight + Cell Populations (wounded mice)
Spleen Weight
0.0901
Weight (mg)
200
150
100
50
Spleen Neutrophil
0.0146
% of CD45+
3
Spleen Neutrophil
0.0146
Spleen Monocyte
0.0128
4
Spleen Ly6Chi Monocyte
0.0505

Supplemental Figure 5: a, Kdm5a$^{fl/fl}$ mice were crossed with Cre-ERT2 to create tamoxifen-inducible knockout mice. Cre+ (Kdm5a-WT) or Cre- (Kdm5a-KO) were fed an HFD over 10 weeks and tamoxifen was injected for 5 days. After two weeks of the final injection of tamoxifen, indicated organs/tissues were harvested for western blotting. Anti-Kdm5a antibody blot (above) and the stain-free image visualizing protein loading (bottom) are shown. **b,** TLR4 median fluorescent intensity of LSK cells after 1-hour LPS treatment (100ng/mL) normalized to average Cre- MFI (n=4 Cre-, 2 Cre+). One-way ANOVA with multiple comparisons (Sidak). **c,** Percentage of blood neutrophils, monocytes, and Ly6C high monocytes 10 days after wounding by dorsal skin biopsy (n=6 Cre-, 4 Cre+). Unpaired t-test. **d,** Spleen weight and percentage of neutrophils, monocytes, and Ly6C high monocytes in the spleen of wounded mice (n=6 Cre-, 4 Cre+). Unpaired t-test.
